# Limitations of microbial iron reduction under extreme conditions

**DOI:** 10.1093/femsre/fuac033

**Published:** 2022-07-16

**Authors:** Sophie L Nixon, Emily Bonsall, Charles S Cockell

**Affiliations:** Department of Earth and Environmental Sciences, Manchester Institute of Biotechnology, University of Manchester, Manchester, M1 7DN, United Kingdom; Biological and Environmental Sciences, University of Stirling, Stirling, FK9 4LA, United Kingdom; UK Centre for Astrobiology, School of Physics and Astronomy, University of Edinburgh, Edinburgh, EH9 3FD, United Kingdom

**Keywords:** extremophiles, microbial iron reduction, thermodynamics, biochemistry, limits to life

## Abstract

Microbial iron reduction is a widespread and ancient metabolism on Earth, and may plausibly support microbial life on Mars and beyond. Yet, the extreme limits of this metabolism are yet to be defined. To investigate this, we surveyed the recorded limits to microbial iron reduction in a wide range of characterized iron-reducing microorganisms (*n* = 141), with a focus on pH and temperature. We then calculated Gibbs free energy of common microbially mediated iron reduction reactions across the pH–temperature habitability space to identify thermodynamic limits. Comparing predicted and observed limits, we show that microbial iron reduction is generally reported at extremes of pH or temperature alone, but not when these extremes are combined (with the exception of a small number of acidophilic hyperthermophiles). These patterns leave thermodynamically favourable combinations of pH and temperature apparently unoccupied. The empty spaces could be explained by experimental bias, but they could also be explained by energetic and biochemical limits to iron reduction at combined extremes. Our data allow for a review of our current understanding of the limits to microbial iron reduction at extremes and provide a basis to test more general hypotheses about the extent to which biochemistry establishes the limits to life.

## Teaser

The authors present a comprehensive review of the observed limits of growth by iron-reducing microorganisms characterized to date, alongside predictions of energetic limits using thermodynamic calculations, and discuss the unexplored regions of the habitability space for this widespread and ancient metabolism, with implications for our understanding of life in the most extreme environments on Earth and the search for life elsewhere.

## Introduction

As the fourth most abundant element in the Earth’s crust, iron is harnessed by a diverse array of microorganisms for energy generation. Microorganisms able to harness energy liberated from iron redox reactions are widespread in time and space, with some iron-reducing microorganisms occupying the deepest roots of the tree of life (Vargas et al. 1998). Although a great deal of research has been carried out on iron-reducing microorganisms (reviewed in Lovley 1987, 1991, 2013, Nealson and Myers 1992, Lloyd 2003, Lovley et al. 2004, Weber et al. 2006), little attention has been paid to microbial iron reduction in extreme environments. The notable exception is a recent review of iron reduction in extreme acidophiles (Malik and Hedrich 2022), reflecting the relative focus on low pH iron-reducing microorganisms.

There are several reasons to better understand the extreme limits to the iron reduction metabolism. First, iron-reducing microorganisms play important roles in mineral, metal, and carbon transformations in the environment (Weber et al. 2006). For example, the majority of iron-reducing microorganisms utilize organic electron donors for iron reduction, and many (e.g. members of the *Geobacteraceae* family) completely oxidize these organic compounds to carbon dioxide (Lovley et al. 2004). As such, iron reducers play a significant role in global carbon cycling, and carbon mineralization in particular (Canfield et al. 1993). Without better understanding of the limits to this metabolism, the contribution of iron-reducing microorganisms to globally significant biogeochemical cycles, especially the global carbon cycle, is likely to be underestimated.

Second, the use of hydrogen as an alternative electron donor to organic compounds in iron-reducing strains from subsurface environments (e.g. *Geobacter hydrogenophilus*; Coates et al. 2001) may have significant implications for short- to medium-term subsurface storage of hydrogen. As such, understanding the diversity and prevalence of hydrogen-utilizing iron reducers could be useful in planning for a net zero emissions future.

Third, owing to the versatility of many iron-reducing microorganisms to use a wide range of organic electron donors and alternative terminal electron acceptors, they have long been seen as useful in the biological remediation of contaminated environments. For example, *Shewanella oneidensis* MR-1 can mediate the reduction of toxic and mobile chromium, Cr(VI), to a less soluble and toxic form, Cr(III) (Myers et al. 2000). Similarly, *Geobacter metallireducens* GS-15 can grow from the reduction of the soluble oxidized form of uranium, U(VI), yielding poorly soluble U(IV) (Lovley et al. 1991). Other iron-reducing microorganisms can use hazardous aromatic organic compounds as electron donors, for instance xylene [e.g. *Desulfitobacterium aromaticivorans* UKTL (Kunapuli et al. 2010)], naphthalene [e.g. *Geomonas terrae* Red111 (Xu et al. 2019)], phenol, toluene, and benzene [e.g. *G. metallireducens* GS-15 (Lovley et al. 1993a)]. Iron-reducing microorganisms are also known to cause environmental issues, such as the mobilization of toxic arsenic into groundwater (Islam et al. 2004). Better understanding microbial iron reduction at extremes may offer new insights and optimized pathways for bioremediation purposes, including in extreme environments. Other applications of iron-reducing microorganisms, including those with electroactive properties that can be harnessed for current generation in microbial fuel cells (reviewed in Logan et al. 2019), could similarly benefit from a focus on extremophiles.

Fourth, given the ubiquity of iron coupled with a lack of oxygen elsewhere in our solar system, microbial iron reduction is considered one of a number of plausible metabolisms to support microbial metabolism beyond Earth. Mars, e.g. hosts a plethora of ferric iron minerals known to serve as terminal electron acceptors to microorganisms on Earth (Nixon et al. 2013). Further, the delivery of organics to the surface of Mars from carbonaceous meteorites may represent a significant supply of potential electron donors, with many meteoritic organics already known to serve as electron donors for iron reduction (Nixon et al. 2012). Our understanding of habitability elsewhere is constrained by our knowledge of life on Earth, and in order to guide the search for life on Mars and other planetary bodies it is crucial to first define the limits to life on Earth.

The environmental limits of life on Earth must ultimately be set by physical limits. One of these limits is the availability of energy in an environment. Any source of energy to support life must be thermodynamically favourable. This establishes one boundary condition to the growth and reproduction of life. Less understood is how biochemical factors impose additional constraints on the limits to life on the planet.  Clearly, we would expect that at very high temperatures at which macromolecules dissociate, e.g. no predicted exothermic Gibbs free energy would make life possible. However, at less extreme conditions, are particular combinations of extremes biochemically incompatible with respect to the adaptations required or are the combined energetic needs in some multiple extremes too great to be met with the energy available, even though in theory the metabolic process itself is thermodynamically favourable?

To address this question, we mapped the physical and chemical limits to microbial iron reduction reported in the literature to predictions of energy available across a range of environmentally relevant conditions. We gathered together a comprehensive set of data of all the characterized strains capable of growth from iron reduction to date (*n* = 141), available in full as a supplementary file. This database covers the conditions for growth, habitats, and location of origin for all strains, and electron donors and acceptors used. Using thermodynamic calculations of Gibbs free energy, we probe the feasibility of the metabolism across the physical and chemical conditions in which they have been found and use this to address two questions: (1) Are the boundaries of iron-reducing life on Earth at the theoretical thermodynamic limits of the iron reduction reaction, or are there extremes that are energetically conductive to iron reduction within which no iron-reducing microorganisms have yet been discovered? (2) Do empty spaces that could theoretically be occupied on account of energetically favourable conditions represent environments that are unfavourable because of biochemical limits, or they are unsampled? In addressing these questions, our aims are to (i) comprehensively assess the known limits to microorganisms that harness energy from iron reduction reactions, (ii) provide an updated summary of the diversity, growth habits, habitats of origin, and substrate utilization for all known iron reducers characterized to date, and (iii) shed light on the general question of the extent to which microbial life at extremes is limited by purely energetic considerations or the underlying limits to life’s biochemical architecture. As a corollary, we also draw attention to the relative paucity of information on extremophilic, and particularly polyextremophilic, strains, suggesting that more systematic and comprehensive data on the distribution of microbial life in extremes, coupled with modelling, would allow us to more effectively discover the factors that limit life at the extremes.

### Survey of iron-reducing microorganisms

We compiled a comprehensive database of all characterized iron-reducing microorganisms (each representing a single species) to date, with a focus on extremophiles. We chose to focus on characterized isolates because type strain publications serve as a source of the broadest and most consistent information on growth conditions, range of electron donors and acceptors used, and habitats of origin, all of which we address here. To the best of our knowledge, these 141 microorganisms represent all characterized isolates (recognized by the International Code of Nomenclature of Prokaryotes) capable of growth by ferric iron reduction as of November 2021. A notable exception is *Geogemma barrossii* Strain 121, the most hyperthermophilic microorganism described to date (Kashefi and Lovley 2003), which is not a fully characterized isolate but is included nonetheless given its relevance to the scope of this study. Some strains were not described with iron-reducing capacity in their original descriptions [e.g. *Pelobacter carbonicolicus* (Schink 1984)], and where relevant we included references for subsequent publications that demonstrate this ability (e.g. Lovley et al. 1995). Conversely, some fermentative microorganisms are known to use iron as a minor electron acceptor during fermentation, yet given that fermentation is the main route for energy acquisition and growth, we have not included them in our database. Great care was taken to generate a fully comprehensive database, i.e. as up to date as possible, but some eligible strains may have been overlooked, and there are doubtless many more that are known to reduce ferric iron but no published account exists, and so they have not been included here. We acknowledge that this approach is necessarily limited and does not incorporate evidence of iron-reducing microorganisms in enrichment cultures and 16S rRNA gene and metagenomic sequencing surveys. As such our assessment is nonexhaustive, but serves as a reliable baseline with which to address the limits to life supported by this metabolism. The full database, with details of all strains included, is available in File S1 (Supporting Information).

Cardinal growth data were used to classify strains based on their adaptation to extremes of temperature, pH, salinity, and pressure. Where optimal growth conditions were expressed as a range (e.g. 30–35°C, pH 7–7.5), the midpoint between the two given values was used (e.g. 32.5°C and pH 7.25, respectively). To assess geographical coverage of strains, locations of samples used for initial enrichment and subsequent isolation given in type strain papers were plotted using coordinates or, where these were lacking, regional location details.

### Phylogenetic analysis

A 16S rRNA gene phylogenetic tree was constructed to represent all genera in the database.  Full-length 16S rRNA genes were obtained from public databases for each strain, and the longest gene sequence for each genus represented in the database was selected to be used in construction of the tree [see File S1 (Supporting Information) for accession numbers]. A total of 64 gene sequences were aligned in Geneious Prime (version 2020.0.3) using the MUSCLE version 3.8.425 using a maximum of eight iterations. Ends of aligned sequences were trimmed to match the shortest sequence, and any alignment column with three bases or less across all sequences was removed. This yielded a final alignment of 1265 nucleotides in length. A phylogenetic tree was constructed from this alignment with RAxML 8.2.11 [general time-reversible (GTR) gamma nucleotide model, 999 bootstrap replicates] within Geneious Prime.

### Calculating thermodynamic limits

All life requires energy to maintain cellular function and power metabolic processes.  We employed Gibbs free energy calculations to define the thermodynamic limits to iron reduction as an assessment of whether this reaction could support microbial growth and reproduction [equations and values used are given in File S2 (Supporting Information)]. For this analysis, we focussed on temperature and pH since these are the most widely reported growth data available for characterized strains with which to compare our predictions. Gibbs free energy for a given reaction provides an indication of whether a reaction is thermodynamically feasible and favourable under a given set of conditions. Where the Gibbs free energy of a reaction is negative in value, the reaction will proceed spontaneously. Where the value is positive, energy input from outside the system is required for the reaction to proceed. A value of zero indicates the reaction is in equilibrium and will not proceed in either forward or reverse reaction. As such, Gibbs free energy of a given reaction must be negative for a redox reaction to be harnessed by microbial life. The magnitude of the Gibbs free energy indicates how far from equilibrium the reaction is, and hence gives an indication of thermodynamic favourability. A more negative Gibbs free energy is indicative of a more favourable reaction than one with a Gibbs free energy value close to zero (Cockell and Nixon 2013).

Here, Gibbs free energy provides an indication of the fundamental energy availability from different iron reduction reactions under a range of conditions. As such, Gibbs free energy predictions can be used to identify baseline energetic limits to iron reduction reactions as a means of powering microbial life, and from these predictions hard limits on habitability can be inferred. In acknowledgement of the energy required for cell survival and maintenance, we imposed a threshold of −20 kJ per electron transferred to determine feasible redox couple reaction conditions. This threshold reflects minimum energy requirements previously postulated across different microbial metabolisms (Schink 1997, Hoehler 2004, 2007). For a redox reaction to be favourable for growth, Gibbs free energy for that reaction under a specified set of conditions must be −20 kJ per electron transferred or lower. Values of between 0 and −20 kJ per electron transferred are considered unfavourable, and those above 0 kJ per electron transferred unfeasible.

The electron donors and acceptor couples considered for these calculations were chosen based on their environmental relevance. Electron donors include acetate and hydrogen, coupled to the reduction of goethite (FeO(OH)), hematite (Fe_2_O_3_), and ferrihydrite (Fe(OH)_3_). Both acetate and hydrogen are common fermentation products that are widely used by iron-reducing strains (File S1, Supporting Information). Few strains in our survey have been shown to reduce goethite, and none have been shown to reduce hematite (File S1, Supporting Information). However, the vast majority of strains have not been tested for iron reduction with crystalline iron oxides, and we include them here because they are the most prevalent ferric iron oxides in the environment (Schwertmann and Cornell. 1991). Although magnetite has been shown to serve as an electron acceptor by some iron-reducing microorganisms (e.g. *Thermincola ferriacetica* Z-0001; Zavarzina et al. 2007), the majority of iron-reducing strains have not been shown to use it, and in fact the microbially mediated reduction of ferric iron oxides often leads to the production of it (e.g. Lovley et al. 1987). For these reasons, magnetite was not included as an electron acceptor in our calculations. We also note that Fe^3+^ may be associated with organic matter as complexes or as, e.g. (oxyhydr)oxide and phosphate minerals, themselves associated with organic material. We have not considered them here. Their redox potentials are poorly known, making it difficult to model them. Better knowledge of the redox behaviour of such complexes would improve the calculations.

To plot thermodynamic favourability across a range of combined pH–T conditions relevant to microbial growth, Gibbs free energy of reaction for the six redox pairs of interest under nonstandard conditions was calculated using Equation (1) as

(1a) $$\begin{eqnarray*}
{\Delta G} = - RT\,\ln \,{K_c} + RT\ln \frac{{a_Y^ya_Z^z}}{{a_B^ba_D^d}},
\end{eqnarray*}$$

where

(1b) $$\begin{eqnarray*}
{K}_c = \frac{{[\rm Y]}^y\, {[\rm Z]}^z}{{[\rm B]}^b\, {[\rm D]}^d},
\end{eqnarray*}$$

for the reaction: *b*B + *d*D = *y*Y + *z*Z. *R* is the gas constant (8.31 J K-1 mol-1), *T* is temperature in Kelvin, and *K_c_* is the equilibrium constant. To compare theoretical limits with observed growth conditions detailed in the database, we considered the temperature range of –10–130°C and pH range from 0 to 14. All Gibbs free energy of reaction values were normalized for electrons transferred in the given redox reaction.

In calculating Gibbs free energy for the six redox couples of interest, a number of assumptions were made. First, we assumed that acetate was completely oxidized to CO_2_ and H_2_O (given as HCO_3_^–^ and H^+^ under the aqueous conditions of these redox reactions). Second, we assumed that the reduction of any ferric iron terminal electron acceptor will lead to the formation of Fe^2+^_(aq)_. In reality, the Fe(II) formed from these reactions is incorporated into minerals, such as magnetite or siderite (e.g. Kashefi and Lovley 2003). However, this is controlled in part by the broader geochemistry of the system, which we are unable to capture in our equations. Third, given that most ferric iron oxides are unstable at low pH conditions, ferric iron was assumed to be present as Fe^3+^_(aq)_ below pH 3, regardless of the electron acceptor used in the redox couple. It is worth noting that the two crystalline forms of iron oxide considered here (goethite and hematite) can be synthesized in the laboratory from ferrihydrite, also included in our calculations. For example, hematite can be formed from ferrihydrite under conditions favourable to ferrihydrite aggregation (at around pH 8) or when the system is dehydrated, whereas goethite can be formed from ferrihydrite at lower temperatures when the pH deviates significantly from circumneutral (Schwertmann and Cornell 1991). However, we have chosen to consider each ferric oxide in isolation in our calculations to avoid imposing yet more assumptions around the exact conditions these transformations might occur. We recognize that these assumptions lead to somewhat generalized calculations since they cannot account for the complexities of real environments. However, it was necessary to impose these assumptions in order to develop a standardized approach allowing for direct comparisons within the pH–temperature parameter space addressed.

### Extremophilic iron-reducing microorganisms are the minority

**Figure 1.**
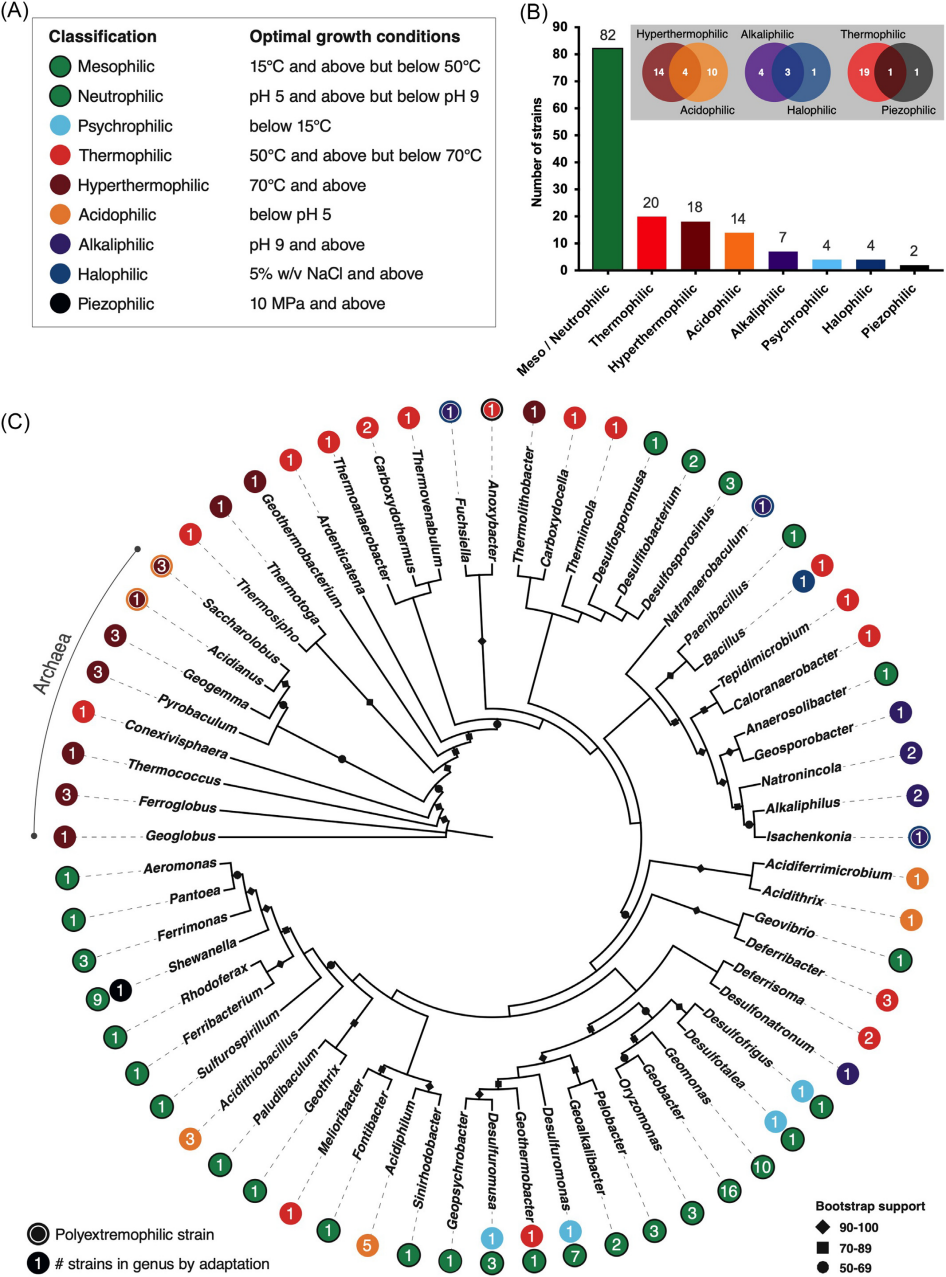
Adaptations and diversity of iron-reducing strains in the databas. **(A)** Classifications used to categorize strains; **(B)** abundance of extremophilic and polyextremophilic (grey box) strains; and **(C)** 16S rRNA gene phylogenetic tree of genera represented in the database, the number of strains per genus (numbered spots) and adaptations of those strains (colours correspond to those in (A). All green (mesophilic/neutrophilic) data points are bordered with black to help distinguish them from red data points.

Based on definitions given in Fig. 1(A), most iron-reducing strains in the database (57%; 80 out of 141) are not classified as extremophiles (Fig. 1). Of those that are (*n* = 61), the highest number are classified as thermophiles (*n* = 20) or hyperthermophiles (*n* = 18). Only four strains are classified as extremophilic with respect to low temperatures; more strains are classified as extremophiles with respect to low (*n* = 14) or high (*n* = 8) pH. A similarly low number of strains are classified as halophilic (*n* = 4). Only two strains are classified as piezophilic (Fig. 1B, Table 1). A total of eight strains are classified as polyextremophilic, where optimal growth occurs at conditions considered extremophilic with respect to more than one parameter. Half of these polyextremophiles are hyperthermophilic and acidophilic (*n* = 4), and represent the only strains capable of growth at combined extremes of pH and temperature. Almost half (*n* = 3) preferentially grow at high salinity and pH. The remaining polyextremophilic strain is adapted to high temperatures and pressures (Fig. 1B). These results suggest that extremophilic iron-reducing microorganisms are rare or less sampled compared to those that preferentially grow in moderate conditions, and polyextremophilic iron-reducers are even less characterized.

**Table 1.** Extremophilic iron-reducing strains and their relevant optimal (and range) growth conditions. Asterisks denote polyextremophilic strains (classified by more than one extremophilic adaptation according to definitions given in Figure 1a). ND = not determined. Taxonomy is based on the SILVA 16S rRNA reference database (release 138).

Adaptation	Strain Name	(Kingdom | Class | Family)	(°C/pH/% w/v NaCl/MPa)	References
Psychrophilic	*Desulfotalea psychrophila* LSv54	*Bacteria* | *Desulfobulbia* | *Desulfocapsaceae*	10 (-1.8 - 19)	Knoblauch et al. (1999)
	*Desulfofrigus oceanense* ASv26	*Bacteria* | *Desulfobacteria* | *Desulfolunaceae*	10 (-1.8 - 16)	Knoblauch et al. (1999)
	*Desulfuromusa ferrireducens* 102	*Bacteria* | *Desulfuromonadia* |*Geopsychrobacteraceae*	14 - 17 (-2 - 23)	Vandieken et al. (2006)
	*Desulfuromonas svalbardensis* 112	*Bacteria* | *Desulfuromonadia* | *Desulfuromonadaceae*	14 (-2 - 20)	Vandieken et al. (2006)
Thermophilic	*Deferrisoma camini* S3R1	*Bacteria* | *Defferrisomatia* | *Defferrisomataceae*	50 (36 - 62)	Slobodkina et al. (2012a)
	*Tepidimicrobium ferriphilum* SB91	*Bacteria* | *Clostridia* |	50 (26 - 62)	Slobodkin et al. (2006a)
	*Geothermobacter ehrlichi* SS015	*Bacteria* | *Desulfuromonadia* | *Geobacteraceae*	55 (35 - 65)	Kashefi et al. (2003)
	*Melioribacter roseus* P3M‐2	*Bacteria* | *Ignavibacteria* | *Melioribacteraceae*	55 (35 - 60)	Podosokorskaya et al. (2013)
	*Thermincola ferriacetica* Z-0001	*Bacteria* | *Clostridia* | *Thermincolaceae*	57 - 60 (45 - 70)	Zavarzina et al. (2007)
	*Carboxydocella manganica* SLM 61	*Bacteria* | *Thermincolia* | C*arboxydocellaceae*	58 - 60 (26 - 70)	Slobodkina et al. (2012b)
	*Deferrisoma paleochoriense* MAG-PB1	*Bacteria* | *Defferrisomatia* | *Defferrisomataceae*	60 (30 - 70)	Perez-Rodriguez et al. (2016)
	*Deferribacter autotrophicus* SL50	*Bacteria* | *Deferribacteres* | *Deferribacteraceae*	60 (25 - 75)	Slobodkina et al. (2009)
	*Deferribacter thermophilus* BMA	*Bacteria* | *Deferribacteres* | *Deferribacteraceae*	60 (50 - 65)	Greene et al. (1997)
	*Deferribacter abyssi* JR	*Bacteria* | *Deferribacteres* | *Deferribacteraceae*	60 (45 - 65)	Miroshnichenko et al. (2003)
	*Caloranaerobacter ferrireducens* DY22619	*Bacteria* | *Clostridia* |	60 (40 - 70)	Zeng et al. (2015)
	*Carboxydothermus ferrireducens* JW/AS-Y7	*Bacteria* | *Desulfotomaculia* | *Carboxydothermaceae*	65 (50 - 74)	Slobodkin et al. (1997), Slobodkin et al. (2006b)
	*Bacillus infernus* TH-23	*Bacteria* | *Bacilli* | *Bacillaceae*	61 (45 - ND)	Boone et al. (1995)
	*Anoxybacter fermentans* DY22613*	*Bacteria* | *Halanaerobiia* | *Halobacteroidaceae*	60 - 62 (44 - 72)	Zeng et al. (2015)
	*Carboxydothermus pertinax* Ug1	*Bacteria* | *Desulfotomaculia* | *Carboxydothermaceae*	65 (50 - 70)	Yoneda et al. (2012)
	*Thermovenabulum gondwanense* R270	*Bacteria* | *Thermovenabulia* |	65 (50 - 70)	Ogg et al. (2010)
	*Conexivisphaera calida* NAS-02	*Archaea* | *Conexivisphaerales* | *Conexivisphaeraceae*	65 (60 - 70)	Kato et al. (2021)
	*Ardenticatena maritima* 110S	*Bacteria* | *Anaerolineae* | *Ardenticatenaceae*	55 - 70 (30 - 75)	Kawaichi et al. (2013)
	*Thermoanaerobacter siderophilus* SR4	*Bacteria* | *Thermoanaerobacteria* | *Thermoanaerobacteraceae*	69 - 71 (39 - 78)	Slobodkin et al. (1999)
	*Thermosipho ferrireducens* JL129W03	*Bacteria* | *Thermotogae* | *Fervidobacteriaceae*	70 (55 - 75)	Chen et al. (2021)
Hyperthermophilic	*Thermolithobacter ferrireducens* JW/KA-2	*Bacteria* | *Clostridia* | *Thermolithobacteraceae*	73 (50 - 75)	Sokolova et al. (2007)
	*Thermococcus indicus* IOH1	*Archaea* | *Thermococci* | *Thermococcaceae*	80 (70 - 82)	Lim et al. (2020)
	*Thermotoga maritima* MSB8	*Bacteria* | *Thermotogae* | *Thermotogaceae*	80 (55 - 90)	Huber et al. (1986), Vargas et al. (1998)
	*Saccharolobus shibatae* B12*	*Archaea* | *Thermoprotei* | *Sulfolobaceae*	81 (55 - 86)	Grogan et al. (1990), Hiroyuki and Kurosawa (2018)
	*Saccharolobus caldissimus* HS-3*	*Archaea* | *Thermoprotei* | *Sulfolobaceae*	85 (65 - 93)	Sakai and Kurosawa (2018)
	*Saccharolobus solfataricus* DSM 1616*	*Archaea* | *Thermoprotei* | *Sulfolobaceae*	87 (50 - 87)	Sakai and Kurosawa (2018), Zilig et al. (1980)
	*Ferroglobus placidus* DSM 10,642	*Archaea* | *Archaeoglobi* | *Archaeoglobaceae*	85 (65 - 95)	Chen et al. (2021), Tor and Lovley (2001), Hafenbradl et al. (1996)
	*Geoglobus ahangari* 234	*Archaea* | *Archaeoglobi* | *Archaeoglobaceae*	88 (65 - 90)	Kashefi et al. (2002a)
	*Geothermobacterium ferrireducens* FW1a	*Bacteria* | *Thermodesulfobacteria* | *Thermodesulfobacteriaceae*	85 - 90 (59 - 101)	Kashefi et al. (2002b)
	*Pyrobaculum ferrireducens* 1860	*Archaea* | *Thermoprotei* | *Thermoproteaceae*	90 - 95 (75 - 98)	Slobodkina et al. (2015)
	*Ferroglobus pacificus* 139	*Archaea* | *Archaeoglobi* | *Archaeoglobaceae*	95 (ND)	Kashefi et al. (2008)
	*Geogemma pacifica* 136	*Archaea* | *Thermoprotei* | *Pyrodictiaceae*	95 (ND)	Kashefi et al. (2008)
	*Ferroglobus indicus* 297	*Archaea* | *Archaeoglobi* | *Archaeoglobaceae*	100 (ND)	Kashefi et al. (2008)
	*Geogemma indica* 296	*Archaea* | *Thermoprotei* | *Pyrodictiaceae*	100 (ND)	Kashefi et al. (2008)
	*Pyrobaculum aerophilum* IM2	*Archaea* | *Thermoprotei* | *Thermoproteaceae*	100 (74 - 102)	Völkl et al. (1993), Lovley ()
	*Pyrobaculum islandicum* DSM 4184	*Archaea* | *Thermoprotei* | *Thermoproteaceae*	100 (75 - 98)	Vargas et al. (1998), Huber et al. (1987), Kashefi and Lovley (2000), Feinberg et al. (2008)
	*Geogemma barossii* 121	*Archaea* | *Thermoprotei* | *Pyrodictiaceae*	105 (85 - 121)	Kashefi and Lovley (2003)
Acidophilic	*Acidianus manzaensis* NA-1	*Archaea* | *Thermoprotei* | *Sulfolobaceae*	1.2 - 1.5 (1.0 - 5.0)	Yoshida et al. (2006)
	*Acidithiobacillus ferriphilus* M20	*Bacteria* | γ-*Proteobacteria* | *Acidithiobacillaceae*	2.0 (1.5 - ND)	Falagán and Johnson (2016)
	*Acidithiobacillus ferrianus MG*	*Bacteria* | γ-*Proteobacteria* | *Acidithiobacillaceae*	2.0 (ND)	Norris et al. (2020)
	*Acidithiobacillus ferrooxidans* ATCC 23,270	*Bacteria* | γ-*Proteobacteria* | *Acidithiobacillaceae*	2.0 - 3.0 (1.3 - 4.5)	Drobner et al. (1990), Das et al. (1992), Pronk et al. (1992), Kelly and Wood (2000)
	*Acidiphilium acidophilum* ATCC 27,807	*Bacteria* | α-*Proteobacteria* | *Acetobacteraceae*	3.0 - 3.5 (1.5 - 6.0)	Hiraishi et al. (1998), Okamura et al. (2015)
	*Acidiferrimicrobium australe* USS-CCA1	*Bacteria* | *Acidimicrobiia* |	3.0 (1.7 - 4.5)	González et al. (2020)
	*Acidiphilium cryptum* JF-5	*Bacteria* | α-*Proteobacteria* | *Acetobacteraceae*	3.0 (2.1 - 5.8)	Küsel et al. (1999)
	*Acidiphilium rubrum* ATCC 35,905	*Bacteria* | α-*Proteobacteria* | *Acetobacteraceae*	3.0 (ND)	Okamura et al. (2015), Wichlacz et al. (1986)
	*Acidithrix ferrooxidans* PY-F3	*Bacteria* | *Acidimicrobiia* | *Acidimicrobiaceae*	3.0 - 3.2 (2.0 - 4.4)	Jones and Johnson (2015)
	*Saccharolobus shibatae* B12*	*Archaea* | *Thermoprotei* | *Sulfolobaceae*	3.0 (1.5 - 6.0)	Grogan et al. (1990), Hiroyuki and Kurosawa (2018)
	*Saccharolobus caldissimus* HS-3*	*Archaea* | *Thermoprotei* | *Sulfolobaceae*	3.0 (1.5 - 6.0)	Sakai and Kurosawa (2018)
	*Acidiphilium multivorum* AIU 301	*Bacteria* | α-*Proteobacteria* | *Acetobacteraceae*	3.2 - 4.0 (1.9 - 5.9)	Wakao et al. (1994)
	*Acidiphilium iwatense* MS8	*Bacteria* | α-*Proteobacteria* | *Acetobacteraceae*	3.5 (2.0 - 5.5)	Okamura et al. (2015)
	*Saccharolobus solfataricus* DSM 1616*	*Archaea* | *Thermoprotei* | *Sulfolobaceae*	4.5 (3.5 - 5.0)	Sakai and Kurosawa (2018), Zilig et al. (1980)
Alkaliphilic	*Isachenkonia alkalipeptolytica* Z-1701*	*Bacteria* | *Clostridia* |	9.0 - 9.3 (7.5 - 10.2)	Zavarzina et al. (2020)
	*Alkaliphilus peptidifermentans* Z-7036	*Bacteria* | *Clostridia* |	9.1 (7.5 - 9.7)	Zhilina et al. (2009)
	*Geosporobacter ferrireducens* IRF9	*Bacteria* | *Clostridia* | *Thermotaleaceae*	9.0 - 9.5 (6.5 - 10.0)	Hong et al. (2015)
	*Natranaerobaculum magadiense* Z-1001*	*Bacteria* | *Natranaerobiia* | *Natranaerobiaceae*	9.25 - 9.5 (7.5 - 10.7)	Zavarzina et al. (2013)
	*Desulfonatronum buryatense* Ki5	*Bacteria* | *Desulfovibrionia* | *Desulfonatronaceae*	9.4 (7.5 - 10.5)	Ryzhmanova et al. (2013)
	*Fuchsiella ferrireducens* Z-7101*	*Bacteria* | *Halanaerobiia* | *Halobacteroidaceae*	9.8 (8.5 - 10.7)	Zhilina et al. (2015)
	*Alkaliphilus namsaraevii* X-07–2	*Bacteria* | *Clostridia* |	10.0 (7.0 - 10.7)	Zakharyuk et al. ()
Halophilic	*Ishachenkonia alkalipeptolytica* Z-1701*	*Bacteria* | *Clostridia* |	12.3 (5.8 - 18.1)	Zhilina et al. (2009)
	*Natranaerobaculum magadiense* Z-1001*	*Bacteria* | *Natranaerobiia* | *Natranaerobiaceae*	7 - 8 (0 - 8.8)	Zavarzina et al. (2013)
	*Fuchsiella ferrireducens* Z-7101*	*Bacteria* | *Halanaerobiia* | *Halobacteroidaceae*	7 (1.1 - 15.8)	Zhilina et al. (2015)
	*Bacillus arsenicoselenatis* E1H	*Bacteria* | *Bacilli* | *Bacillaceae*	6 (2 - 12)	Switzer Blum et al. (1998)
Piezophilic	*Shewanella profunda* LT13a	*Bacteria* | γ-*Proteobacteria* | *Shewanellaceae*	10 (0.1 - 50)	Toffin et al. (2004)
	*Anoxybacter fermentans* DY22613*	*Bacteria* | *Halanaerobiia* | *Halobacteroidaceae*	20 (0.1 - 55)	Zeng et al. (2015)

The strains included in the database exhibit high phylogenetic diversity (Fig. 1C). Most strains belong to bacteria, regardless of classification (Fig. 1C). Of the archaeal strains in the database (16 of 141 strains), all but one [*Conexivisphaera calida* NAS-02 (TACK group); Kato et al. 2021] are classified as hyperthermophiles (Fig. 1C and Table 1). Of the 63 genera represented in the database, most contain three or fewer strains (Fig.   1C), further highlighting the diversity of microorganisms capable of iron reduction. Despite this diversity, the names given to these genera are often not indicative of iron-reducing capabilities (e.g. *Bacillus, Aeromonas*, and *Caloranaerobacter*). Indeed, many names are instead indicative of sulfur metabolism, for instance *Desulfuromonas, Desulfosporosinus*, and *Desulfonatronum* (Fig. 1C and Table 1). Such names can be misleading, and strains belonging to these genera that are traditionally considered sulfate-reducing bacteria have previously been thought not to have true dissimilatory iron-reducing capabilities (Lovley 2013). Their inclusion in the database presented here, however, highlights otherwise.

Genera with the greatest number of strains in the database include *Geobacter* (*n* = 16) and *Shewanella* (*n* = 10), both widely known for their ability to reduce ferric iron and other metals (e.g. Lloyd 2003). However, only one strain in these two genera is classified as extremophilic: *Shewanella profunda* LT13a (Toffin et al. 2004), a piezophile with an optimum growth pressure of 20 MPa, and an upper pressure growth limit of 55 MPa. These results highlight that the extremophilic iron-reducing microorganisms characterized to date are not taxa that are conventionally associated with dissimilatory iron reduction, but in fact span a diverse range of phylogenetic groups (Fig. 1C and Table 1).

Of the extremophiles identified in our survey, relatively more is understood about acidophilic iron reduction than other extreme conditions investigated here. Although a relatively small number of characterized isolates are classified as acidophilic (*n* = 14), this metabolic trait is actually widespread among acidophilic iron-oxidizing microorganisms, and hence we acknowledge that this number is in fact misleading. It has been noted previously that the majority of acidophilic iron oxidizers capable of using electron donors other than ferrous iron are also capable of iron reduction at low pH (Johnson et al. 2012), and though a handful of these are captured in our survey [e.g. *Acidithiobacillus ferrooxidans* (Pronk et al. 1991), *Acidiferrimicrobium australe* (González et al. 2020)] there are many more that are not (e.g. Johnson et al. 2012).

### Evidence of biogeographical sampling bias

**Figure 2.**
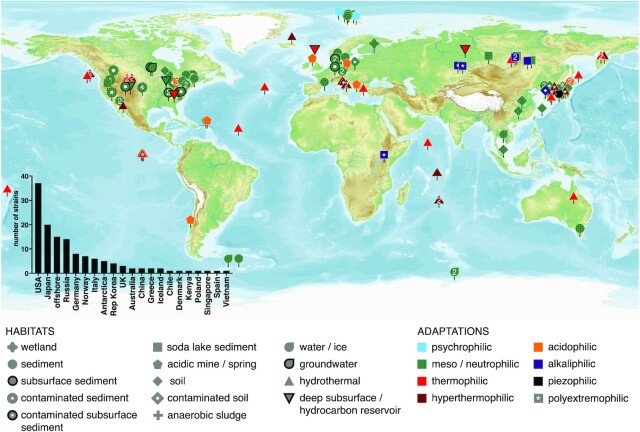
Origin and habitat type of characterized iron-reducing strains, classified by adaptation (see Fig. 1(A) for definitions). The inset barchart indicates the number of strains isolated by country/territory of origin. All green (mesophilic/neutrophilic) data points have a dotted pattern to distinguish them from red data points.

The iron-reducing strains characterized to date are not only phylogenetically diverse, but originate from a wide diversity of habitat types, including wetlands, anoxic sludge, contaminated land, and the deep subsurface (Fig. 2). The most common among these are marine (*n* = 19) and freshwater (*n* = 10) sediments, marine (*n* = 20) and terrestrial (*n* = 15), and hydrothermal systems and soil (*n* = 14). Of the seven strains that could not be plotted in Fig. 2 due to lack of relevant location data, two strains derive from biological hosts, both belonging to the *Shewanella* genus; *S. olga* OK-1, which was isolated from the surface of red algae (Nozue et al. 1992) and *S. pealeana* ANG-SQ1, isolated from the nidamental gland of a squid (Leonardo et al. 1999). *Aeromonas hydrophila* was isolated from a tin of milk with a fishy odour (Knight and Blakemore 1998), while four strains were isolated from microbial fuel cells [*Fontibacter ferrireducens* Z-7101 (Zhang et al. 2013), *Geobacter anodireducens* SD-1 (Sun et al. 2014), *Geopsychrobacter electrodiphilus* A1 (Holmes et al. 2004), and *Sinorhodobacter ferrireducens* SgZ-3 (Yang et al.^2013^)].

Despite the wide diversity of habitat type of origin, Fig. 2 highlights biogeographical bias. For instance, a large number of strains were isolated from the USA (*n* = 37), as well as Japan (*n* = 20), Russia (*n* = 14), and Germany (*n* = 8). There are large swathes of the globe from which no known iron-reducing strains have been isolated. For instance, only one strain has been isolated from the African and South American continents, only two from Australasia (both from Australia) and none from the Middle East. A relatively small number of strains have been isolated from high latitudes, with all four psychrophilic strains deriving from Svalbard, and a similar number deriving from the Antarctic continent. Surprisingly, no iron-reducing microorganisms have been isolated from Greenland, despite reports of viable cold-adapted iron-reducing microorganisms in enrichment experiments from glacial environments (Nixon et al. 2017). We note that a large number of strains whose optimal growth temperatures classify them as mesophilic are in fact psychrotolerant (*n* = 41), with a number of these originating from high latitudes (Fig. 2). The geographical bias evident in Fig. 2 is best explained by proximity of these field locations to research groups that routinely enrich for, and isolate, iron-reducing microorganisms, leading to publication of type strain papers used to compile the database presented here.

There is some evidence of targeted searches for iron-reducing microorganisms from extreme environments, such as hydrothermal systems (*n* = 35), polar environments (*n* = 11), and soda lakes (*n* = 10; Fig. 2). Clearly, the isolation of novel iron-reducing strains from a chosen environment is driven by the underlying research questions, which often focus on the bioremediation potential of contaminated land, and the use of iron-reducing microorganisms in microbial fuel cells. The geographical distribution of characterized strains to date indicates that the relatively small number of extremophilic iron reducers can be explained by a lack of focus on extreme environments (Fig. 2). It is, therefore, likely that iron-reducing strains capable of growth at more extreme conditions than are represented here reside in yet-unexplored extreme environments on Earth.

### Growth by iron reduction occurs in a pH–T habitability ‘sweet spot’

**Figure 3.**
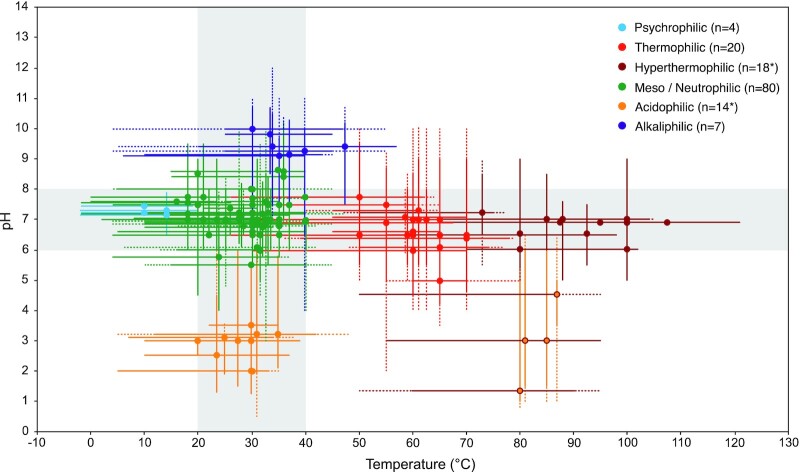
pH–temperature habitability parameter space of iron-reducing strains in the database. Each point represents optimal growth conditions with respect to temperature (T) and pH. Solid error bars represent the range in growth T and pH conditions for each strain, and dashed error bars represent the range of T and pH conditions tested for each strain. All points are colour-coded according to their pH and T adaptation classification (see Fig. 1A). The grey box highlights the ‘Goldilocks zone’ of relatively narrow pH (6–8) and T (20–40°C) growth optima that almost all strains (135 of 141) fall within.

We plotted optimal and ranges of growth conditions for each strain in the database with respect to pH and temperature (Fig. 3). These parameters are by far the most widely reported growth conditions in type strain papers. The majority of strains (*n* = 93) have optimal growth temperatures between 20 and 40°C. Similarly, most strains (*n* = 113) grow optimally between pH 6 and 8. In fact, all but six strains grow optimally within one or both of these relatively narrow ranges (grey shading, Fig. 3). Temperatures conducive to iron reduction range from −2°C [*Desulfuromonas svalbardensis* 112 and *Desulfuromusa ferrireducens* 102 (Vandieken et al. 2006)] to 121°C (*Geogemma barossii* strain 121; Kashefi and Lovley 2003), but are broadly restricted to circumneutral pH. Similarly, iron-reducing microorganisms can grow within a wide range of pH, from pH 1.3 [*A. ferrooxidans* ATCC 23270 (Drobner et al. 1990)] to pH 10.7 [*Fuchsiella ferrireducens* IRF9 (Zhilina et al. 2015) and *Alkaliphilus namsaraevii* X-07–1 (Zakharyuk et al. 2017)], but within the mesophilic temperature range of 20–40°C.

The six strains that are the exceptions to this rule include *Natranaerobaculum magadiense* Z-1001, with growth optima of pH 9.25–9.5 and 45–50°C (Zavarzina et al. 2013), *C. calida* NAS-02 (pH 5 and 60°C; Kato et al. 2021), and the hyperthermophilic acidophiles belonging to the genera *Saccharolobus* (Sakai and Kurosawa 2018) and *Acidianus* (Yoshida et al. 2006, Fig. 3). These latter four polyextremophilic strains, which stand alone in Fig. 3 as outliers, all belong to the *Archaea*, and demonstrate that the combined high temperature and low pH region of the parameter space is conducive to the iron reduction metabolism. Indeed, *A. manzaensis* NA-1 represents the most acidophilic strain in our survey, with a minimum growth pH of 1.0 (Table 1, Fig. 3; Yoshida et al. 2006).

### Electron donor use is generally not affected by extremophilic classification

**Figure 4.**
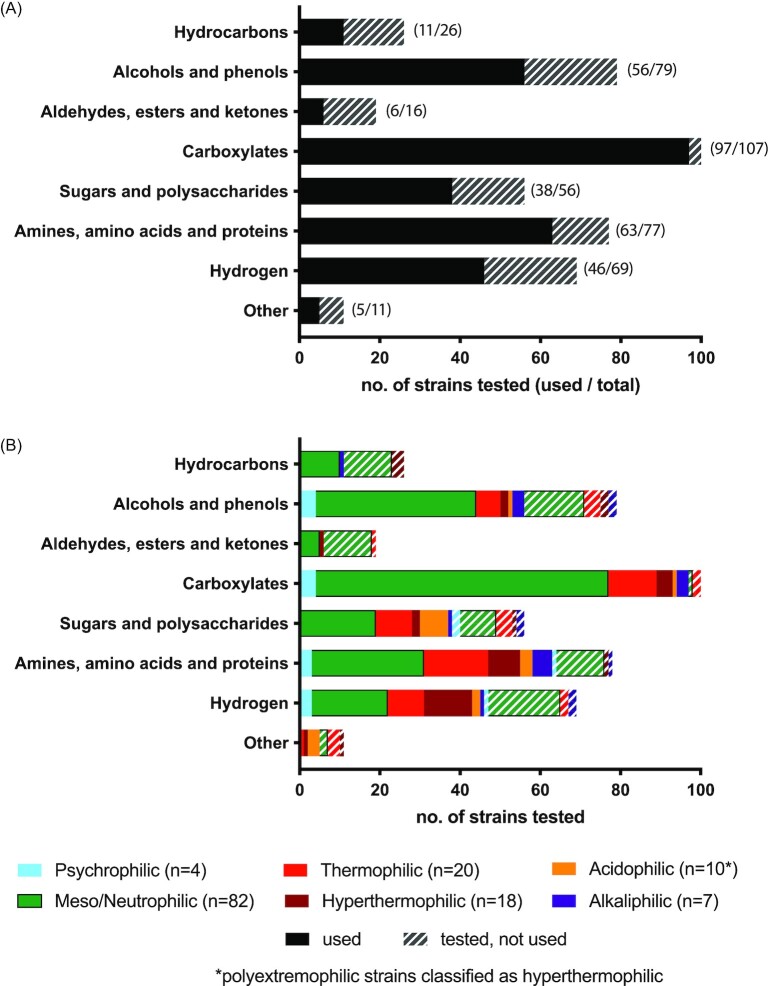
Electron donor use by all strains in the database **(A)** and grouped by adaptation **(B)**. Solid colour indicates the electron donor is used for iron reduction, cross-hatched colour indicates tested but not used. Numbers in parentheses indicate the number of strains that can use an electron donor in that group compared with the total number of strains tested. ‘Other’ includes carbon monoxide and elemental sulfur. Green (mesophilic/neutrophilic) bars have a black border to distinguish them from red bars.

We assessed the range of electron donors used for the microbial reduction of iron [dissolved, poorly crystalline, and crystalline forms, see File S1 (Supporting Information)] amongst strains in the database and whether substrate use differs among extremophiles compared with nonextremophilic strains (Fig. 4).  We were driven by the question of whether the energy demand to operate in extreme conditions restricts the range or type of electron donors available for iron reduction. It is clear from Fig. 4(A) that the range of compound classes tested as electron donors varies substantially from strain to strain, with the majority of all strains tested for the use of carboxylates (e.g. acetate, lactate, propionate, fumarate, and succinate) compared to just 11% of strains tested for use of aldehydes, esters, and ketones (e.g. benzaldehyde and *p*-hydroxybenzaldehyde). Despite these inconsistencies, it is clear that most iron-reducing strains tested for the use of carboxylate compounds are able to couple these to iron reduction (Fig. 4A). Alcohol and phenol compounds (particularly ethanol, propanol, glycerol, and butanol) are the next most widely used electron donors, with over two-thirds of tested strains showing use, followed by amines, amino acids and proteins, and hydrogen. When assessed based on temperature and pH growth range (Fig. 4B), there is little evidence to suggest extremophiles have a restricted range of electron donor use, with the corresponding numbers of strains able or unable to use different electron donors mirroring the overall trends. Notable exceptions are hydrogen; all hyperthermophiles tested for its use could couple its oxidation to iron reduction for growth (Fig. 4B; File S1, Supporting Information), in line with previous reports that the use of hydrogen as an electron donor for iron reduction is widespread among thermophiles (Lovley et al. 2004). Furthermore, amines, amino acids and proteins appear to be more commonly used by thermophiles, and the only strains known to use elemental sulfur are acidophiles (Fig. 4B; File S1, Supporting Information). We, therefore, conclude that, while extremophilic class does not generally affect electron donor use, extremophilic strains appear to draw on inorganic electron donors more than nonextremophilic strains.

### Thermodynamic limits to iron reduction metabolism

**Figure 5.**
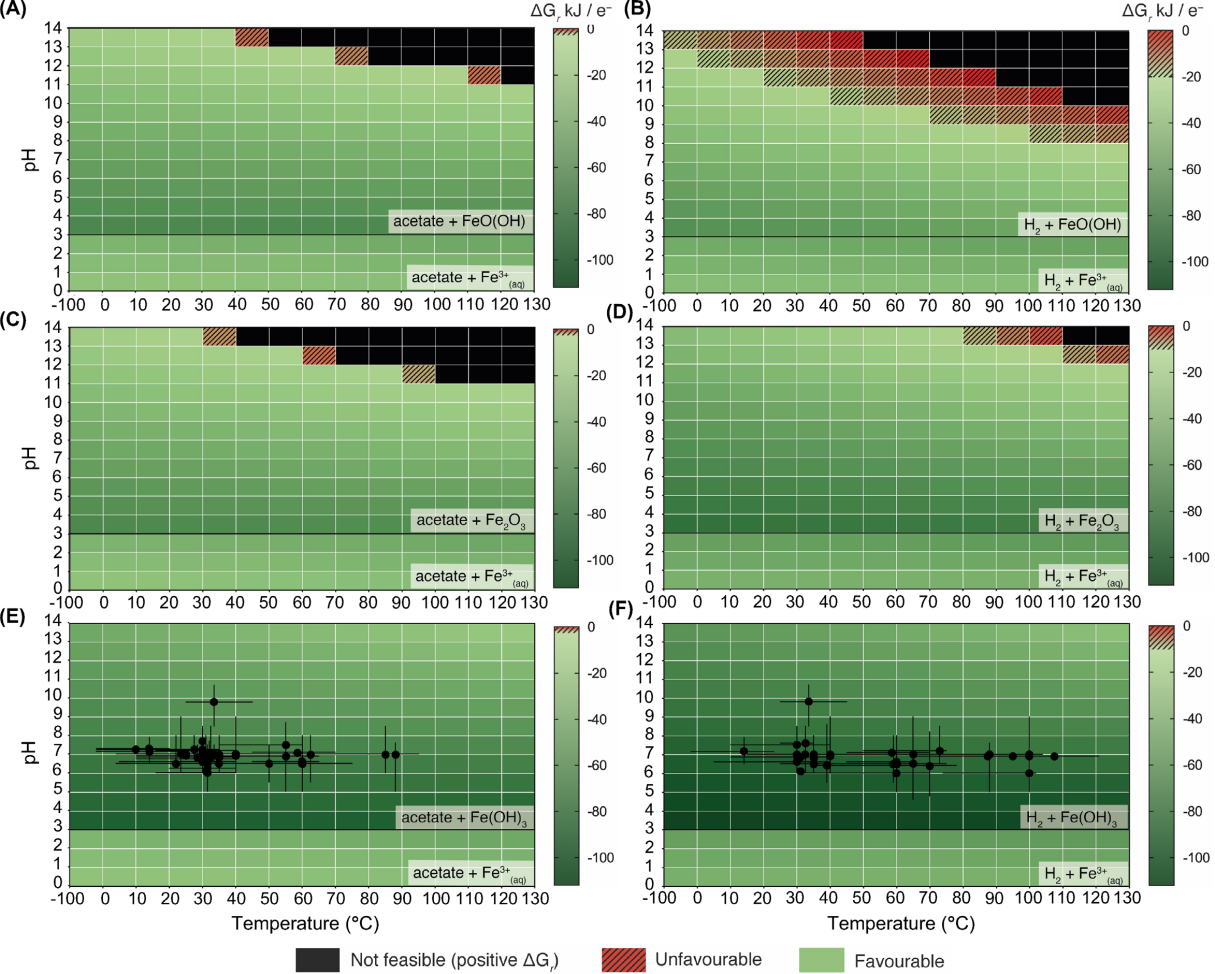
Gibbs free energy of reaction (ΔGr) for iron reduction reactions known to support growth by strains in the database across the pH–temperature habitability space, expressed in kJ per electron transferred for the following iron reduction reactions considered: goethite (FeO(OH)) with acetate **(A)** and hydrogen **(B)**; hematite (Fe_2_O_3_) with acetate **(C)** and hydrogen **(D)**; and ferrihydrite (Fe(OH)_3_) with acetate **(E)** and hydrogen **(F)**. In all cases, the ferric iron phase is assumed to be aqueous (Fe^3+^(aq)) below pH3, regardless of redox couple and temperature. Cells in green are considered favourable for growth by iron reduction; cells in patterned red are considered favourable enough for survival and maintenance only but unfavourable for growth (calculated as −20 kJ per electron transferred for the given reaction); black cells represent positive ΔGr and, therefore, indicate thermodynamically infeasible reactions at the associated conditions. Strains that have been shown to grow from these iron reduction redox couples are overlain on corresponding heatmaps.

We calculated Gibbs free energy of reaction for six environmentally relevant iron reduction redox couples across the range of pH and temperature conditions discussed above, and overlaid corresponding growth data for strains in our database known to use these redox couples (Fig. 5). For hydrogen coupled to ferrihydrite, the most favourable redox couple considered here, there are no regions of this pH–temperature habitability space where this reaction is thermodynamically unfavourable (Fig. 5F). The same is true for acetate coupled to ferrihydrite (Fig. 5E), an environmentally widespread combination, although the energetic yields are lower than with hydrogen at the same pH and temperature conditions. The next most favourite coupling is hematite coupled to hydrogen (Fig. 5D), followed by goethite coupled to acetate (Fig. 5A); hematite coupled to acetate (Fig. 5C), with goethite coupled to hydrogen representing the least energetically favourable per electron transferred (Fig. 5B). The two ferrihydrite redox couples considered here are the only redox couples that are known to support growth by iron reduction by strains in the database (Fig. 5E and C).

Our survey highlights the low number of strains that have been tested with crystalline iron oxide electron acceptors regardless of electron donor. Of the eight strains tested for goethite reduction, *Anoxybacter fermentans* DY22613, *Caloranaerobacter ferrireducens* DY22619 (Zeng et al. 2015) and *Thermosipho ferrireducens* JL129W03 (Chen et al. 2021) have been shown to couple its reduction to complex electron donors, such as yeast extract, peptone, and glucose (File S1, Supporting Information). *Shewanella alga* strain BrY is also capable of growth by coupling hydrogen oxidation to goethite reduction (Roden and Zachara 1996; note strain OK-1 of this species was included in our database, hence BrY is not plotted in Figs 3 or 5). A total of eight strains were also tested for ability to use hematite as the terminal electron acceptor, but none were capable of iron reduction. In agreement with prior research, these limitations seem not to be thermodynamic (Fig. 5A–D). Indeed, prior research indicates surface area, active site availability, crystalline disorder and microheterogeneities affect the ability of *S. alga* to utilize crystalline ferric iron phases (Roden and Zachara 1996, Roden and Urrutia 1999, Urrutia et al. 1998). Cutting et al. (2009) showed that synthetic iron oxides of a range of crystallinities can be reduced by *Geobacter sulfurreducens* (growth was not directly measured), but more crystalline phases such as hematite and goethite were subject to substantially less reduction than poorly crystalline phases. Despite these known limitations, our survey highlights that goethite can support growth of some iron-reducing microorganisms, and our calculations indicate that these redox couples are favourable over a wide range of environmental conditions considered here. Experimental bias away from testing crystalline phases is evident from our survey and warrants further study.

Regardless of electron donor–acceptor couple, the trend for most favourable conditions for electron donors coupled to ferric iron oxides is towards combinations of low pH and temperature, and the least favourable or unfeasible conditions occur at high pH and temperature combinations. The trend is reversed for all electron donors coupled with aqueous ferric iron, e.g. where pH is below 3. However, all combinations of low pH conditions across the full range of temperatures are thermodynamically favourable (Fig. 5). Based on the predictions shown across the pH–T space in Fig. 5, the overall most and least favourable iron reduction reactions considered are hydrogen with ferrihydrite (Fig. 5A) and hydrogen with goethite (Fig. 5D), respectively.

**Figure 6.**
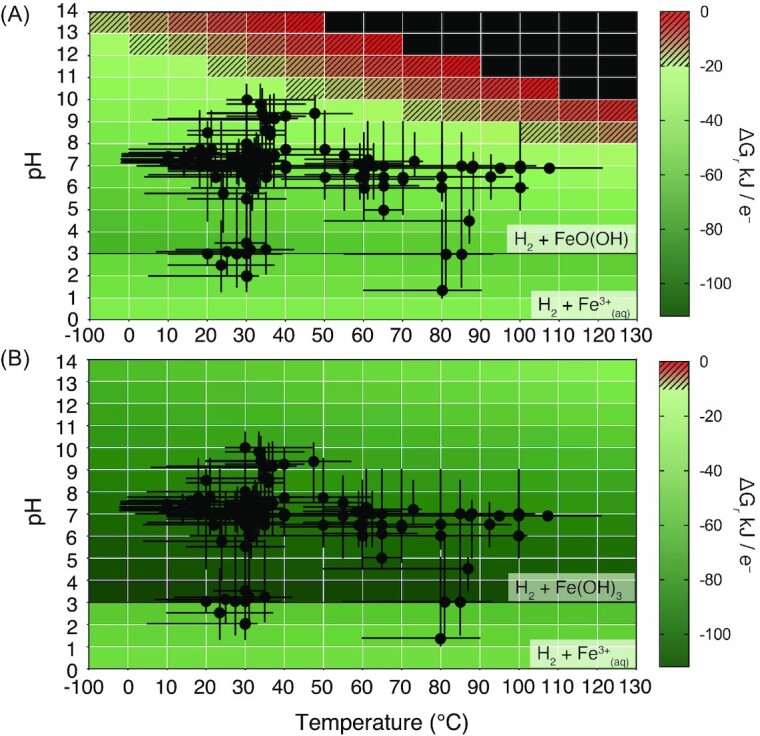
Observed pH and temperature growth conditions plotted with thermodynamic predictions for the most **(A)** and least **(B)** favourable redox couples across the parameter space. In both cases, the ferric iron phase is assumed to be aqueous (Fe^3+^(aq)) below pH 3, regardless of redox couple and temperature. Cells in green are considered favourable for growth by iron reduction; cells in patterned red are considered favourable enough for survival and maintenance, but not growth (calculated as −20 kJ per electron transferred for the given reaction); black cells represent positive ΔGr and, therefore, indicate thermodynamically infeasible reactions at the associated conditions.

To compare theoretical predictions with observed limits of microbial iron reduction, we plotted the cardinal growth data of all strains summarized in Fig. 3 over the theoretical predictions for the overall most and least thermodynamically favourable relevant redox couples across this parameter space (Fig. 6). In doing so, we addressed the hypothesis that reported optima and limits of growth by microbial iron reduction conform to predicted limits to thermodynamic favourability.  This comparison demonstrates broad consistencies with the observed and predicted results. Most notably, in the case of least favourable redox couple (hydrogen with goethite), no optimal growth conditions are plotted in regions of the habitability space with thermodynamically unfavourable Gibbs free energies of reaction (Fig. 6A). One strain, *F. ferrireducens* Z-7101 (Zhilina et al. 2015), is capable of growth up to pH 10.7 at 45°C, a thermodynamically unfavourable combination according to the Gibbs free energy threshold of −20 kJ per e- transferred imposed on these calculations. However, consistent with the theoretical limits, no other maximal or minimal growth conditions occupy thermodynamically unfavourable or infeasible parameter spaces (Fig. 6). As such, the combined results suggest that the combination of high pH and temperature is a fundamental limitation to iron reduction reactions (though high temperature combined with low pH is not), and may explain the lack of strains from environments characterized by these combined conditions.

Inconsistent with observed data, however, is the predicted trend of most favourable conditions for iron reduction. According to Gibbs free energy calculations, the theoretically most favourable regions of all considered redox couples are pH 3 and −10°C (Fig. 5), yet these conditions are not known to support growth by any iron-reducing microorganisms in the database, or indeed (to the best of our knowledge) any microbial metabolism. As highlighted in Fig. 3, the majority of iron-reducing strains cluster with circumneutral pH and moderate temperatures (the habitability ‘sweet spot’). The obvious explanation for the contrasting trends in terms of thermodynamic favourability is that the Gibbs free energy calculations employed here are over-simplistic, and do not incorporate kinetic and physiological limitations to iron-reduction metabolism. Instead, the theoretical trends are simply a reflection of the balanced equation representing the redox reaction in question. Where the terminal electron acceptor is a ferric oxide [e.g. acetate coupled to hematite; Equation (2)], the hydrogen ions are on the left of the equilibrium. According to Le Chatelier’s principle, a decrease in pH (increase in concentration of H^+^) favours the forward reaction, yielding a more negative Gibbs free energy of reaction. Since we have assumed the electron acceptor is a ferric oxide for all pH–T conditions above pH 3, the dominant trend for all redox couples is one of increased favourability with decreased pH. In contrast, where the ferric iron electron acceptor is in the aqueous phase (below pH 3 in our calculations), the hydrogen ions are on the other side of the equilibrium, yielding a less negative Gibbs free energy as the pH decreases [Equation (3)].

(2) $$\begin{eqnarray*}
4{\rm F{e}_2{O}_3} + {\rm C{H}_3CO{O}}^- + 15{\rm H}^ + \Longleftrightarrow {\rm 8F{e}}^{2 + } + {\rm 8{H}_2O} + {\rm 2HC{O}_3}^-
.
\end{eqnarray*}$$

(3) $$\begin{eqnarray*}
{\rm 8Fe}^{3+} + {\rm CH_{3}COO}^- + {\rm 4H_2O} \Longleftrightarrow {\rm 8Fe}^{2 +} + {\rm 2HCO}_{3}^- + {\rm 9H}^+
.
\end{eqnarray*}$$

These inconsistencies between predicted and observed habitable conditions for iron-reducing microorganisms, and ways in which the limits of this metabolism can be challenged further, are discussed below.

### The knowledge gap

In this work, we created a comprehensive database of isolated and characterized iron-reducing microorganisms and their physiological limits. We used it to address some fundamental hypotheses about the limits to iron reduction as a microbial metabolism, while also providing an updated view of the diversity, growth habits, and biogeography of all characterized iron reducers described in the literature to date. In so doing, this work raises general questions about the limits to life in extremes and what establishes the growth and reproductive limits to the microbial biosphere.

This study shows that the currently described strains of iron-reducing microorganisms in the pH–T space do not fill the habitable space predicted by thermodynamic calculations of energy availability. Instead, they cluster in a distinctive pattern along isolated pH and temperature extremes, but not in regions where these two extremes are combined. To our knowledge, there are only four known isolates capable of growth by iron reduction that serve as exceptions to this rule, three of which belong to the *Saccharolobus* genus and the other to the *Acidianus* genus of Archaea. There are several possible explanations for the discrepancies between the experimental isolate data and theoretical thermodynamic data presented here. We suggest ways in which they can be disentangled and experimentally addressed.

(1) Our thermodynamic calculations may not be accurate. We have drawn attention to the assumptions we made (such as the lack of organic complexants and the presence of ferric iron in aqueous form below pH 3). Other limitations include the assumption that ferric iron will be reduced solely to Fe2+ in aqueous form, when in reality the stability and fate of Fe2+ produced will depend on environmental conditions (e.g. precipitation as carbonates at high pH). This in turn will influence the energetic yields of the iron reduction reaction. Furthermore, the values of the redox potentials of the Fe(III) minerals or Fe(III) in associated with organic complexes are not well-defined and depend on many factors such as particle size, presence of defects at the mineral surface, association of the mineral surface with natural organic matter, with phosphate and other ions, and so on. Better data on redox potentials would improve the capacity of theoretically model the potentially habitable space. Finally, we have isolated iron reduction reactions from other geochemical cycles to simplify our assessment, yet we acknowledge that iron geochemistry is intrinsically linked to other cycles in nature, most notably the sulfur cycle. These other geochemical processes may serve as sinks for otherwise available ferric iron (for instance the presence of sulfide) or the ferrous iron produced in microbially mediated iron reduction (such as ferrous phosphate and carbonate precipitation). However, even if these values of redox potentials were improved and other limitations addressed, they might alter the values across the whole pH–T space, but they would not be expected to lead to a prediction of the cross-shaped distribution observed in the isolated strains. Therefore, we do not think that thermodynamic assumptions explain this distribution. Nonetheless, improved thermodynamic modelling would certainly improve the confidence of the absolute limits to microbial iron reduction that we identify.

(2) There may be other energetic limits which our thermodynamic calculations do not consider. For example, the reduction of ferric iron electron acceptors that are in mineral form may be kinetically limited. An example could be the high pH-induced crystallization of ferrihydrite, a readily available electron acceptor, to magnetite, leading to a kinetically less available (or indeed for most strains an unavailable) electron acceptor for iron reduction. In this case, high pH conditions could be less favourable to microbial iron reduction than thermodynamic considerations would suggest. The opposite is known to be true in low pH environments, where the greater solubility of ferric iron at pH 3 and below facilitates the use of ferric iron as an electron acceptor (Johnson et al. 2012). This hypothesis of kinetic limitations could be tested by carrying out more laboratory experiments to study the availability of electron donors and acceptors at the edges of the thermodynamic limits to map geochemical and kinetic limits to iron reduction as an additional ‘layer’ restricting this process at extremes.

(3) The observations suggest bias in the isolation of strains. This bias could stem from two sources: field or experimental bias. First, as we have shown in this work, there is a geographical bias in sampling. However, there is no obvious reason why extremophile researchers would have deliberately focussed on neutral pH and extreme temperature environments or mesophilic environments with extremes of pH. However, combined extremes of pH and temperature do require particular environments (such as low pH and high temperatures in volcanic geothermal springs), and it may be that these combined extremes have not been sufficiently explored for iron reducing isolates. This limitation could be addressed by field work specifically focussed on isolating iron-reducing organisms from the empty regions we identify and launching field expeditions to collect appropriate samples for isolation, particularly in environments with combined extreme conditions. For example, we note the growing range of databases that focus on comprehensive studies of extreme environments such as the one thousand springs project in New Zealand to investigate geothermal springs (https://1000springs.org.nz/). Such databases offer powerful resources for identifying specific locations with the desired extreme combinations. Indeed, the construction of diagrams like Fig. 6 can be used to rationally direct sampling efforts in Earth’s extreme environments to explore the underlying factors that limit life at the extremes. The inclusion of four acidophilic hyperthermophiles in our survey demonstrates that this combined set of extreme conditions does not preclude microbial iron reduction, yet the same cannot be said of other pH and temperature combinations without further targeted study.

Second, another potential source of sampling bias could be in the laboratory. If researchers have run experiments where the temperature is maintained constant and the pH varied and *vice versa* without a systematic study of the combinations of these two factors, then the cross-shaped distribution we observe will result. This sampling bias hypothesis is indeed supported by the data in Fig. 3, which show the growth ranges tested during the characterization of the isolates. In general, combinations of extremes are not tested by researchers. We suggest that the value of isolate-characterization work would be greatly enhanced if researchers would carry out growth analyses at different combinations of extremes and not focus on the extremes of individual stressors. Even better, researchers should challenge isolates with combinations of extremes that deliberately explore the regions near thermodynamic limits.

Another reason for suspecting that the data we present suffer from sampling bias is that some reported experiments provide geochemical and 16S rRNA gene sequencing evidence suggesting the presence of iron-reducing microorganisms in environments from which isolates have not been obtained and characterized. For example, iron reduction has been observed in high pH (> 11) microcosms simulating low level nuclear waste disposal sites (Byrd et al. 2021), which is potentially attributed to members of the Firmicutes.  In our study, we focussed on isolates because we needed to map well defined growth ranges. However, experiments such as those by Byrd et al. (2021) show that there is likely a gap between indirect observations of iron reduction using culture-independent analyses and the isolate data we present here, with culture-independent analysis suggesting that iron reduction occurs in a wider range of conditions than the isolate data would suggest. An example would be general greater difficulty of culturing organisms at high pH compared to low pH due to solubility problems with compounds such as carbonates, problems compounded at low temperatures, potentially limiting isolate data at low T and high pH. More in-depth study and isolation of halophiles at such extremes would assist in removing the sampling bias. At least some of the gaps in Fig. 6, therefore, probably represent sampling bias which must be filled with more study of isolates.

(4) The pattern we observe might show that biochemical factors play a strong role in limiting iron reduction in extremes. There are two possible sources of these limits. First, the theoretically available energy must meet the energetic needs of cell maintenance, repair, and reproduction in the combined extremes. These requirements may exceed these needs, even though the Gibbs free energy is above the theoretically suggested −20 kJ per electron transferred minimum. This hypothesis could be addressed by a more detailed study of the cellular energetic requirements at extreme conditions for the known biochemistry of iron reduction. The various energetic demands of microbial iron reducers at different combined extremes would have to be combined and compared with the theoretical Gibbs free energy to explore how multiple extremes limit this process. Second, energetic considerations aside, there may be a fundamental biochemical limit to life in combined extremes. For example, if some cellular adaptation to cope with low temperatures (such as a more fluid membrane) was incompatible with maintaining a proton motive force (i.e. intracellular pH control) then we could imagine that biochemistry would limit iron reduction in certain combined extremes. To address this hypothesis, we would need to investigate the required biochemical adaptations in combined extremes and determine whether they might place a biochemical limit to life, and in particular whether those biochemical limits might be linked to the specific metabolic machinery required (iron reduction in this case). *A priori*, this hypothesis does not seem to be supported by the evidence, since we find noniron-reducing organisms in pH and temperature extremes where in Fig. 6 we have empty spaces (such as at high pH and temperature; Sarkar 1991, Li et al. 1993, Pikuta et al. 2000), so unless iron reduction causes a specific biochemical limit resulting from the particular molecular machinery required for that metabolism, the empty spaces in Fig. 6 do not seem to be caused by fundamental biomolecular limitations as such.

### Concluding remarks

Our data provide a review of microbial iron reduction at extremes. They also raise general questions about the factors that limit microbial life at extremes, which are still poorly understood. These data allow us to disentangle the variety of factors that might set an ultimate limit on iron reduction. We have suggested how further experimentation might be used to address each of these factors.  Because of the intensive focus that has been given to this metabolism, iron reduction provides a particularly useful workhorse for investigating the limits of life. The data show that we still have some way to go to understand properly what ultimately limits life in addition to the thermodynamic limit, particularly in multiple extremes, but we are much closer to a better grasp of those limits.

## Supplementary Material

fuac033_Supplemental_Files

## References

[bib1] Boone DR , Liu Y, Zhao Z-J *et al*. *Bacillus infernus* sp. nov., an Fe(III)- and Mn(IV)-Reducing Anaerobe from the Deep Terrestrial Subsurface. Int J Syst Evol Microbiol. 1995;45:441–8. 10.1099/00207713-45-3-441 8590670

[bib2] Byrd N , Lloyd JR, Small JS et al. Microbial degradation of citric acid in low level radioactive waste disposal: impact on biomineralization reactions. Front Microbiol. 2021;12:723. 10.3389/fmicb.2021.565855 PMC8114274 33995289

[bib3] Canfield D E , Jørgensen BB, Fossing H et al. Pathways of organic carbon oxidation in three continental margin sediments. Mar Geol. 1993;113:27–40. 11539842 10.1016/0025-3227(93)90147-n

[bib4] Chen Y , He Y, Shao Z et al. *Thermosipho ferrireducens* sp.nov., an anaerobic thermophilic iron(III)-reducing bacterium isolated from a deep-sea hydrothermal sulfide deposits. Int J Syst Evol Microbiol. 2021;71. DOI: 10.1099/ijsem.0.004929. 10.1099/ijsem.0.004929 34328826

[bib107_1659912840212] Coates JD , Bhupathiraju VK, Achenbach LA et al. Geobacter hydrogenophilus, Geobacter chapelli and Geobacter grbiciae, three new, strictly anaerobic, dissimilatory Fe(III)-reducers. Int J Syst Evol Microbiol. 2001;51:581–8. 11321104 10.1099/00207713-51-2-581

[bib5] Cockell CS , Nixon S. The boundaries of lif e. In: Smith I, Leach S, Cockell C (eds), Astrobiology and Astrochemistry: Physical Chemistry in Action. London: Springer, 2013.

[bib108_1659913421780] Cutting RS , Coker VS, Fellowes JW et al. Mineralogical and morphological constraints on the reduction of Fe(III) minerals by Geobacter sulfurreducens. Geochimica et Cosmochimica Acta. 2009;73:4004–22.

[bib109] Das A , Mishra AK, Roy P. Anaerobic growth on elemental sulfur using dissimilar iron reduction by autotrophic Thiobacillus ferrooxidans. FEMS Microbiol Lett. 1992;97:167–72.

[bib7] Drobner E , Huber H, Stetter KO. *Thiobacillus ferrooxidans*, a facultative hydrogen oxidizer. Appl Environ Microbiol. 1990;56:2922–3. 2275538 10.1128/aem.56.9.2922-2923.1990 PMC184866

[bib110] Falagán C , Johnson DB. *Acidithiobacillus ferriphilus* sp. nov., a facultatively anaerobic iron- and sulfur-metabolizing extreme acidophile. Int J Syst Evol Microbiol. 2016;66:206–11. 26498321 10.1099/ijsem.0.000698 PMC4806540

[bib111] Feinberg LF , Srikanth R, Vachet RW et al. Constraints on Anaerobic Respiration in the Hyperthermophilic Archaea *Pyrobaculum islandicum* and *Pyrobaculum aerophilum*. Appl Environ Microbiol. 2008;74:396–402. 18039820 10.1128/AEM.02033-07 PMC2223247

[bib10] González D , Huber KJ, Tindall B et al. *Acidiferrimicrobium australe* gen. nov., sp. nov., an acidophilic and obligately heterotrophic, member of the actinobacteria that catalyses dissimilatory oxido-reduction of iron isolated from metal-rich acidic water in chile. Int J Syst Evol Microbiol. 2020;70:3348–54. 32375942 10.1099/ijsem.0.004179

[bib112] Greene AC , Patel BKC, Sheehy AJ. *Deferribacter thermophilus* gen. nov., sp. nov., a Novel Thermophilic Manganese- and Iron-Reducing Bacterium Isolated from a Petroleum Reservoir. Int J Syst Evol Microbiol. 1997;47:505–9. 10.1099/00207713-47-2-505 9103640

[bib6] Grogan D , Palm P, Zillig W. Isolate B12, which harbours a virus-like element, represents a new species of the archaebacterial genus *Sulfolobus, Sulfolobus shibatae*, sp. nov. Arch Microbiol. 1990;154:594–9. 1703758 10.1007/BF00248842

[bib113] Hafenbradl D , Keller M, Dirmeier R *et al*. *Ferroglobus placidus* gen. nov., sp. nov., a novel hyperthermophilic archaeum that oxidizes Fe2+ at neutral pH under anoxic conditions. Arch Microbiol. 1996;166:308–14. 8929276 10.1007/s002030050388

[bib8] Hiraishi A , Nagashima KVP, Matasuura K et al. Phylogeny and photosynthetic features of *Thiobacillus acidophilus* and related acidophilic bacteria: its transfer to the genus *Acidiphilium* as *Acidiphilium acidophilum* comb. nov. Int J Syst Evol Microbiol. 1998;48:1389–98. 10.1099/00207713-48-4-1389 9828441

[bib15] Hoehler TM . An energy balance concept for habitability. Astrobiology. 2007;7:824–39. 18163865 10.1089/ast.2006.0095

[bib16] Hoehler TM . Biological energy requirements as quantitative boundary conditions for life in the subsurface. Geobiology. 2004;2:205–15.

[bib17] Holmes D E , Nicoll JS, Bond DR et al. Potential role of a novel psychrotolerant member of the family Geobacteraceae, *Geopsychrobacter electrodiphilus* gen. nov., sp. nov., in electricity production by a marine sediment fuel cell. Appl Environ Microbiol. 2004;70:6023–30. 15466546 10.1128/AEM.70.10.6023-6030.2004 PMC522133

[bib9] Hong H , Kim S-J, Min U-G *et al*. *Geosporobacter ferrireducens* sp. nov., an anaerobic iron-reducing bacterium isolated from an oil-contaminated site. Antonie Van Leeuwenhoek. 2015;107:971–7. 25663026 10.1007/s10482-015-0389-3

[bib11] Huber R , Kristjansson JK, Stetter KO. *Pyrobaculum* gen. nov., a new genus of neutrophilic, rod-shaped archaebacteria from continental solfataras growing optimally at 100°C. Arch Microbiol. 1987;149:95–101.

[bib114] Huber R , Langworthy TA, König H *et al*. *Thermotoga maritima* sp. nov. represents a new genus of unique extremely thermophilic eubacteria growing up to 90°C. Arch Microbiol. 1986;144:324–33.

[bib21] Islam FS , Gault AG, Boothman C et al. Role of metal-reducing bacteria in arsenic release from Bengal Delta sediments. Nature. 2004;430:68–71. 15229598 10.1038/nature02638

[bib22] Johnson DB , Kanao T, Hedrich S. Redox transformations of iron at extremely low pH: fundamental and applied aspects. Front Microbiol. 2012;3:96. 22438853 10.3389/fmicb.2012.00096 PMC3305923

[bib12] Jones RM , Johnson DB. *Acidithrix ferrooxidans* gen. nov., sp. nov.; a filamentous and obligately heterotrophic, acidophilic member of the Actinobacteria that catalyzes dissimilatory oxido-reduction of iron. Res Microbiol. 2015;166:111–20. 25638020 10.1016/j.resmic.2015.01.003

[bib116] Kashefi K , Holmes D E, Baross JA et al. Thermophily in the Geobacteraceae: *Geothermobacter ehrlichii* gen. nov., sp. nov., a Novel Thermophilic Member of the Geobacteraceae from the “Bag City” Hydrothermal Vent. Appl Environ Microbiol. 2003;69:2985–93. 12732575 10.1128/AEM.69.5.2985-2993.2003 PMC154550

[bib14] Kashefi K , Holmes D E, Reysenbach A et al. Use of Fe(III) as an Electron Acceptor To Recover Previously Uncultured Hyperthermophiles: Isolation and Characterization of *Geothermobacterium ferrireducens* gen. nov., sp. nov. Appl Environ Microbiol. 2002a;68:1735–42. 11916691 10.1128/AEM.68.4.1735-1742.2002 PMC123901

[bib27] Kashefi K , Lovley D R. Extending the upper temperature limit for life. Science. 2003;301:934. 12920290 10.1126/science.1086823

[bib13] Kashefi K , Lovley D R. Reduction of Fe(III), Mn(IV), and Toxic Metals at 100°C by *Pyrobaculum islandicum*. Appl Environ Microbiol. 2000;66:1050–6. 10698770 10.1128/aem.66.3.1050-1056.2000 PMC91941

[bib117] Kashefi K , Shelobolina ES, Elliott WC et al. Growth of Thermophilic and Hyperthermophilic Fe(III)-Reducing Microorganisms on a Ferruginous Smectite as the Sole Electron Acceptor. Appl Environ Microbiol. 2008;74:251–258. 17981937 10.1128/AEM.01580-07 PMC2223214

[bib115] Kashefi K , Tor JM, Holmes D E *et al*. *Geoglobus ahangari* gen. nov., sp. nov., a novel hyperthermophilic archaeon capable of oxidizing organic acids and growing autotrophically on hydrogen with Fe(III) serving as the sole electron acceptor. Int J Syst Evol Microbiol. 2002b;52:719–28. 12054231 10.1099/00207713-52-3-719

[bib30] Kato S , Ohnishi M, Nagamori M et al. *Conexivisphaera calida* gen.nov., sp. nov., a thermophilic sulfur- and iron-reducing archaeon,and proposal of Conexivisphaeraceae fam. nov., Conexivisphaeralesord. nov., and Conexivisphaeria class. nov. in thephylum Thaumarchaeota. Int J Syst Evol Microbiol. 2021;71. DOI: 10.1099/ijsem.0.004595. 10.1099/ijsem.0.004595 33295866

[bib18] Kawaichi S , Ito N, Kamikawa R *et al*. *Ardenticatena maritima* gen. nov., sp. nov., a ferric iron- and nitrate-reducing bacterium of the phylum ‘Chloroflexi’ isolated from an iron-rich coastal hydrothermal field, and description of Ardenticatenia classis nov. Int J Syst Evol Microbiol. 2013;63:2992–3002. 23378114 10.1099/ijs.0.046532-0

[bib19] Kelly DP , Wood AP. Reclassification of some species of *Thiobacillus* to the newly designated genera *Acidithiobacillus* gen. nov., *Halothiobacillus* gen. nov. and *Thermithiobacillus* gen. nov. Int J Syst Evol Microbiol. 2000;50:511–6. 10758854 10.1099/00207713-50-2-511

[bib33] Knight V V , Blakemore R. Reduction of diverse electron acceptors by *Aeromonas hydrophila*. Arch Microbiol. 1998;169:239–48. 9477259 10.1007/s002030050567

[bib20] Knoblauch C , Sahm K, Jørgensen BB. Psychrophilic sulfate-reducing bacteria isolated from permanently cold Arctic marine sediments: description of *Desulfofrigus oceanense* gen. nov., sp. nov., *Desulfofrigus fragile* sp. nov., *Desulfofaba gelida* gen. nov., sp. nov., *Desulfotalea psychrophila* gen. nov., sp. nov. and *Desulfotalea arctica* sp. nov. Int J Syst Evol Microbiol. 1999;49:1631–43. 10.1099/00207713-49-4-1631 10555345

[bib35] Kunapuli U , Jahn MK, Lueders T et al. *Desulfitobacterium aromaticivorans* sp. nov. and *Geobacter toluenoxydans* sp. nov., iron-reducing bacteria capable of anaerobic degradation of monoaromatic hydrocarbons. Int J Syst Evol Microbiol. 2010;60:686–95. 19656942 10.1099/ijs.0.003525-0

[bib118] Küsel K , Dorsch T, Acker G et al. Microbial reduction of Fe(III) in acidic sediments: isolation of *Acidiphilium cryptum* JF-5 capable of coupling the reduction of Fe(III) to the oxidation of glucose. Appl Environ Microbiol. 1999;65:3633–40. 10427060 10.1128/aem.65.8.3633-3640.1999 PMC91545

[bib37] Leonardo MR , Moser DP, Barbieri E et al. *Shewanella pealeana* sp. nov., a member of the microbial community associated with the accessory nidamental gland of the squid *Loligo pealei*. Int J Syst Evol Microbiol. 1999;49:1341–51. 10.1099/00207713-49-4-1341 10555311

[bib38] Li Y , Mandelco L, Wiegel J. Isolation and characterization of a moderately thermophilic anaerobic alkaliphile, *Clostridium paradoxum* sp. nov. Int J Syst Evol Microbiol. 1993;43:450–60.

[bib119] Lim JK , Kim YJ, Yang J-A *et al*. *Thermococcus indicus* sp. nov., a Fe(III)-reducing hyperthermophilic archaeon isolated from the Onnuri Vent Field of the Central Indian Ocean ridge. J Microbiol. 2020;58:260–7. 32239454 10.1007/s12275-020-9424-9

[bib40] Lloyd JR Microbial reduction of metals and radionuclides. FEMS Microbiol Rev. 2003;27:411–25. 12829277 10.1016/S0168-6445(03)00044-5

[bib41] Logan BE , Rossi R, Ragab A et al. Electroactive microorganisms in bioelectrochemical systems. Nat Rev Microbiol. 2019;17:307–19. 30846876 10.1038/s41579-019-0173-x

[bib43] Lovley D , Giovannoni SJ, White DC et al. *Geobacter metallireducens* gen. nov. sp. nov., a microorganism capable of coupling the complete oxidation of organic compounds to the reduction of iron and other metals. Arch Microbiol. 1993a;159:336–44. 8387263 10.1007/BF00290916

[bib23] Lovley D . Dissimilatory Fe(III)- and Mn(IV)-Reducing Prokaryotes. In: Rosenberg E, DeLong EF, Lory S et al. (eds.), The Prokaryotes: Prokaryotic Physiology and Biochemistry. Berlin, Heidelberg: Springer Berlin Heidelberg, 2013, 287–308.

[bib46] Lovley DR , Holmes D E, Nevin KP. Dissimilatory Fe(iii) and Mn(iv) reduction. Adv Microb Physiol. 2004;49:219–86. 15518832 10.1016/S0065-2911(04)49005-5

[bib47] Lovley DR , Phillips EJ, Lonergan DJ et al. Fe(III) and S0 reduction by *Pelobacter carbinolicus*. Appl Environ Microbiol. 1995;61:2132–8. 7793935 10.1128/aem.61.6.2132-2138.1995 PMC167486

[bib48] Lovley DR , Phillips EJP, Gorby YA et al. Microbial reduction of uranium. Nature. 1991;350:413–6.

[bib50] Lovley DR , Stolz JF, Nord Jr GL et al. Anaerobic production of magnetite by a dissimilatory iron-reducing microorganism. Nature. 1987;330:252–4.

[bib45] Lovley DR . Dissimilatory Fe(iii) and Mn(iv) reduction. Microbiol Rev. 1991;55:259–87. 1886521 10.1128/mr.55.2.259-287.1991 PMC372814

[bib44] Lovley DR . Organic matter mineralization with the reduction of ferric iron: a review. Geomicrobiol J. 1987;5:375–99.

[bib51] Malik L , Hedrich S. Ferric iron reduction in extreme acidophiles. Front Microbiol. 2022;12:818414. 35095822 10.3389/fmicb.2021.818414 PMC8790237

[bib24] Miroshnichenko ML , Slobodkin AI, Kostrikina NA *et al*. *Deferribacter abyssi* sp. nov., an anaerobic thermophile from deep-sea hydrothermal vents of the Mid-Atlantic Ridge. Int J Syst Evol Microbiol. 2003;53:1637–41. 13130062 10.1099/ijs.0.02673-0

[bib53] Myers CR , Carstens BP, Antholine WE et al. Chromium(VI) reductase activity is associated with the cytoplasmic membrane of anaerobically grown *Shewanella putrefaciens* MR-1. J Appl Microbiol. 2000;88:98–106. 10735248 10.1046/j.1365-2672.2000.00910.x

[bib54] Nealson KH , Myers CR. Microbial reduction of manganese and iron: new approaches to carbon cycling. Appl Environ Microbiol. 1992;58:439–43. 1610166 10.1128/aem.58.2.439-443.1992 PMC195266

[bib55] Nixon SL , Cockell CS, Tranter M. Limitations to a microbial iron cycle on mars. Planet Space Sci. 2012;72. DOI: 10.1016/j.pss.2012.04.003.

[bib56] Nixon SL , Cousins CR, Cockell CS. Plausible microbial metabolisms on mars. Astron Geophys. 2013;54.DOI: 10.1093/astrogeo/ats034.

[bib57] Nixon SL , Telling JP, Wadham JL et al. Viable cold-tolerant iron-reducing microorganisms in geographically diverse subglacial environments. Biogeosciences. 2017;14. DOI: 10.5194/bg-14-1445-2017.

[bib25] Norris PR , Falagán C, Moya-Beltrán A *et al*. *Acidithiobacillus ferrianus* sp. nov.: an ancestral extremely acidophilic and facultatively anaerobic chemolithoautotroph. Extremophiles. 2020;24:329–37. 31980944 10.1007/s00792-020-01157-1 PMC7040056

[bib59] Nozue H , Hayashi T, Hashimoto Y et al. Isolation and characterization of *Shewanella alga* from human clinical specimens and emendation of the description of *S. alg*a. Simidu *et al*., 1990, 335. Int J Syst Microbiol. 1992;42:628–34. 10.1099/00207713-42-4-628 1390113

[bib26] Ogg CD , Greene AC, Patel BKC. *Thermovenabulum gondwanense* sp. nov., a thermophilic anaerobic Fe(III)-reducing bacterium isolated from microbial mats thriving in a Great Artesian Basin bore runoff channel. Int J Syst Evol Microbiol. 2010;60:1079–84. 19666811 10.1099/ijs.0.009886-0

[bib120] Okamura K , Kawai A, Wakao N *et al*. *Acidiphilium iwatense* sp. nov., isolated from an acid mine drainage treatment plant, and emendation of the genus *Acidiphilium*. Int J Syst Evol Microbiol. 2015;65:42–8. 25273513 10.1099/ijs.0.065052-0

[bib28] Pérez-Rodríguez I , Rawls M, Coykendall DK *et al*. *Deferrisoma palaeochoriense* sp. nov., a thermophilic, iron(III)-reducing bacterium from a shallow-water hydrothermal vent in the Mediterranean Sea. Int J Syst Evol Microbiol. 2016;66:830–6. 26610851 10.1099/ijsem.0.000798

[bib63] Pikuta E , Lysenko A, Suzina N et al. *Desulfotomaculum alkaliphilum* sp. nov., a new alkaliphilic, moderately thermophilic, sulfate-reducing bacterium. Int J Syst Evol Microbiol. 2000;50:25–33. 10826784 10.1099/00207713-50-1-25

[bib29] Podosokorskaya OA , Kadnikov V V, Gavrilov SN et al. Characterization of *Melioribacter roseus* gen. nov., sp. nov., a novel facultatively anaerobic thermophilic cellulolytic bacterium from the class Ignavibacteria, and a proposal of a novel bacterial phylum Ignavibacteriae. Environ Microbiol. 2013;15:1759–71. 23297868 10.1111/1462-2920.12067

[bib121] Pronk JT , de Bruyn JC, Bos P et al. Anaerobic Growth of *Thiobacillus ferrooxidans*. Appl Environ Microbiol. 1992;58:2227–30. 16348735 10.1128/aem.58.7.2227-2230.1992 PMC195759

[bib66] Pronk JT , Liem K, Bos P et al. Energy transduction by anaerobic ferric iron respiration in *Thiobacillus ferrooxidans*. Appl Environ Microbiol. 1991;57:2063–8. 16348526 10.1128/aem.57.7.2063-2068.1991 PMC183522

[bib67] Roden EE , Urrutia MM. Ferrous iron removal promotes microbial reduction of crystalline iron(III) oxides. Environ Sci Technol. 1999;33:1847–53.

[bib68] Roden EE , Zachara JM. Microbial reduction of crystalline iron(III) oxides: influence of oxide surface area and potential for cell growth. Environ Sci Technol. 1996;30:1618–28.

[bib31] Ryzhmanova Y , Nepomnyashchaya Y, Abashina T et al. New sulfate-reducing bacteria isolated from Buryatian alkaline brackish lakes: description of *Desulfonatr onum buryatense* sp. nov. Extremophiles. 2013;17:851–9. 23881259 10.1007/s00792-013-0567-z

[bib70] Sakai HD , Kurosawa N. *Saccharolobus caldissimus* gen. nov., sp. nov., a facultatively anaerobic iron-reducing hyperthermophilic archaeon isolated from an acidic terrestrial hot spring, and reclassification of *Sulfolobus solfataricus* as *Saccharolobus solfataricus* comb. nov. and *Sulfolobus shibatae* as *Saccharolobus shibatae* comb. nov. Int J Syst Evol Microbiol. 2018;68:1271–8. 29485400 10.1099/ijsem.0.002665

[bib71] Sarkar A . Isolation and characterization of thermophilic, alkaliphilic, cellulose-degrading *Bacillus thermoalcaliphilus* sp. nov. from termite (*Odontotermes obesus*) mound soil of a semiarid area. Geomicrobiol J. 1991;9:225–32.

[bib72] Schink B . Energetics of syntrophic cooperation in methanogenic degradation. Microbiol Mol Biol Rev. 1997;61:262–80. 9184013 10.1128/mmbr.61.2.262-280.1997 PMC232610

[bib73] Schink B . Fermentation of 2,3-butanediol by *Pelobacter carbinolicus* sp. nov. and *Pelobacter propionicus* sp. nov., and evidence for propionate formation from C2 compounds. Arch Microbiol. 1984;137:33–41.

[bib74] Schwertmann U , Cornell RM. Iron Oxides in the Laboratory: Preparation and Characterization. Weinheim: VCH, 1991.

[bib32] Slobodkin A , Reysenbach A, Strutz N *et al*. *Thermoterrabacterium ferrireducens* gen. nov., sp. nov., a Thermophilic Anaerobic Dissimilatory Fe(III)-Reducing Bacterium from a Continental Hot Spring. Int J Syst Evol Microbiol. 1997;47:541–7. 10.1099/00207713-47-2-541 9103646

[bib34] Slobodkin AI , Sokolova TG, Lysenko AM et al. Reclassification of *Thermoterrabacterium ferrireducens* as *Carboxydothermus ferrireducens* comb. nov., and emended description of the genus *Carboxydothermus*. Int J Syst Evol Microbiol. 2006a;56:2349–51. 17012560 10.1099/ijs.0.64503-0

[bib123] Slobodkin AI , Tourova TP, Kostrikina NA *et al*. *Tepidimicrobium ferriphilum* gen. nov., sp. nov., a novel moderately thermophilic, Fe(III)-reducing bacterium of the order Clostridiales. Int J Syst Evol Microbiol. 2006b;56:369–72. 16449442 10.1099/ijs.0.63694-0

[bib122] Slobodkin AI , Tourova TP, Kuznetsov BB *et al*. *Thermoanaerobacter siderophilus* sp. nov., a novel dissimilatory Fe(III)-reducing, anaerobic, thermophilic bacterium. Int J Syst Evol Microbiol. 1999;49:1471–8. 10.1099/00207713-49-4-1471 10555328

[bib36] Slobodkina GB , Kolganova T V, Chernyh NA *et al*. *Deferribacter autotrophicus* sp. nov., an iron (III) - reducing bacterium from a deep-sea hydrothermal vent. Int J Syst Evol Microbiol. 2009;59:1508–12. 19502344 10.1099/ijs.0.006767-0

[bib39] Slobodkina GB , Lebedinsky A V, Chernyh NA *et al*. *Pyrobaculum ferrireducens* sp. nov., a hyperthermophilic Fe(III)-, selenate- and arsenate-reducing crenarchaeon isolated from a hot spring. Int J Syst Evol Microbiol. 2015;65:851–6. 25510975 10.1099/ijs.0.000027

[bib124] Slobodkina GB , Panteleeva AN, Sokolova TG et al. Carboxydocella manganica sp. nov., a thermophilic, dissimilatory Mn(IV)- and Fe(III)-reducing bacterium from a Kamchatka hot spring. Int J Syst Evol Microbiol. 2012a;62:890–4. 21642487 10.1099/ijs.0.027623-0

[bib125] Slobodkina GB , Reysenbach A-L, Panteleeva AN *et al*. *Deferrisoma camini* gen. nov., sp. nov., a moderately thermophilic, dissimilatory iron(III)-reducing bacterium from a deep-sea hydrothermal vent that forms a distinct phylogenetic branch in the Deltaproteobacteria. Int J Syst Evol Microbiol. 2012b;62:2463–8. 22140176 10.1099/ijs.0.038372-0

[bib126] Sokolova T , Hanel J, Onyenwoke RU et al. Novel chemolithotrophic, thermophilic, anaerobic bacteria *Thermolithobacter ferrireducens* gen. nov., sp. nov. and *Thermolithobacter carboxydivorans* sp. nov. Extremophiles. 2007;11:145–57. 17021657 10.1007/s00792-006-0022-5

[bib84] Sun D , Wang A, Cheng S et al. *Geobacter anodireducens* sp. nov., an exoelectrogenic microbe in bioelectrochemical systems. Int J Syst Evol Microbiol. 2014;64:3485–91. 25052395 10.1099/ijs.0.061598-0

[bib127] Switzer J , Allana B, Bindi B et al. *Bacillus arsenicoselenatis* sp. nov., and *Baci llus selenitireducens*, sp. nov.: two haloalkaliphiles from Mono Lake, California that respire oxyanions of selenium and arsenic. Arch Microbiol. 1998;171:19–30. 9871015 10.1007/s002030050673

[bib86] Toffin L , Bidault A, Pignet P et al. *Shewanella profunda* sp. nov., isolated from deep marine sediment of the Nankai trough. Int J Syst Evol Microbiol. 2004;54:1943–9. 15545415 10.1099/ijs.0.03007-0

[bib128] Tor JM , Lovley DR. Anaerobic degradation of aromatic compounds coupled to Fe(III) reduction by *Ferroglobus placidus*. Environ Microbiol. 2001;3:281–7. 11359514 10.1046/j.1462-2920.2001.00192.x

[bib88] Urrutia MM , Roden EE, Fredrickson JK et al. Microbial and surface chemistry controls on reduction of synthetic Fe(iii) oxide minerals by the dissimilatory iron-reducing bacterium *Shewanella alga*. Geomicrobiol J. 1998:15:269–91.

[bib89] Vandieken V , Mussmann M, Niemann H et al. *Desulfuromonas svalbardensis* sp. nov. and *De sulfuromusa ferrireducens* sp. nov., psychrophilic, Fe(III)-reducing bacteria isolated from Arctic sediments, Svalbard. Int J Syst Evol Microbiol. 2006;56:1133–9. 16627667 10.1099/ijs.0.63639-0

[bib90] Vargas M , Kashefi K, Blunt-Harris EL et al. Microbiological evidence for Fe(iii) reduction on early Earth. Nature. 1998;395:65–7. 9738498 10.1038/25720

[bib129] Völkl P , Huber R, Drobner E *et al*. *Pyrobaculum aerophilum* sp. nov., a novel nitrate-reducing hyperthermophilic archaeum. Appl Environ Microbiol. 1993;59:2918–2926. 7692819 10.1128/aem.59.9.2918-2926.1993 PMC182387

[bib130] Wakao N , Nagasawa N, Matsuura T *et*al. *Acidiphilium multivorum* sp. noc., an acidophilic chemoorganotrophic bacterium from pyritic acid mine drainage. J Gen Appl Microbiol. 1994;40:143–59.

[bib93] Weber K , Achenbach L, Coates JD. Microorganisms pumping iron: anaerobic microbial iron oxidation and reduction. Nat Rev Microbiol. 2006;4:752–64. 16980937 10.1038/nrmicro1490

[bib131] Wichlacz PL , Unz RF, Langworthy TA. *Acidiphilium angustum* sp. nov., *Acidiphilium facilis* sp. nov., and *Acidiphilium rubrum* sp. nov.: Acidophilic Heterotrophic Bacteria Isolated from Acidic Coal Mine Drainage. Int J Syst Evol Microbiol. 1986;36:197–201.

[bib95] Xu Z , Masuda Y, Itoh H et al. *Geomonas oryzae* gen. nov., sp. nov., *Geomonas edaphica* sp. nov., *Geomonas ferrireducens* sp. nov., *Geomonas terrae* sp. nov., four ferric-reducing bacteria isolated from paddy soil, and reclassification of three species of the genus *Geobacter* as members of the genus *Geomonas* gen. nov. Front Microbiol. 2019;10:2201. 31608033 10.3389/fmicb.2019.02201 PMC6773877

[bib96] Yang G , Chen M, Zhou S et al. *Sinorhodobacter ferrireducens* gen. nov., sp. nov., a non-phototrophic iron-reducing bacterium closely related to phototrophic *Rhodobacter* species. Antonie Van Leeuwenhoek. 2013;104:715–24. 23907520 10.1007/s10482-013-9979-0

[bib132] Yoneda Y , Yoshida T, Kawaichi S *et al*. *Carboxydothermus pertinax* sp. nov., a thermophilic, hydrogenogenic, Fe(III)-reducing, sulfur-reducing carboxydotrophic bacterium from an acidic hot spring. Int J Syst Evol Microbiol. 2012;62:1692–7. 21908679 10.1099/ijs.0.031583-0

[bib98] Yoshida N , Nakasato M, Ohmura N et al. *Acidianus manzaensis* sp. nov., a novel thermoacidophilic archaeon growing autotrophically by the oxidation of H2 with the reduction of Fe3+. Curr Microbiol. 2006;53:406–11. 17066338 10.1007/s00284-006-0151-1

[bib133] Zakharyuk A , Kozyreva L, Ariskina E *et al*. *Alkaliphilus namsaraevii* sp. nov., an alkaliphilic iron- and sulfur-reducing bacterium isolated from a steppe soda lake. Int J Syst Evol Microbiol. 2017;67:1990–5. 28632119 10.1099/ijsem.0.001904

[bib100] Zavarzina DG , Sokolova TG, Tourova TP et al. *Thermincola ferriacetica* sp. nov., a new anaerobic, thermophilic, facultatively chemolithoautotrophic bacterium capable of dissimilatory Fe(iii) reduction. Extremophiles. 2007;11:1–7. 16988758 10.1007/s00792-006-0004-7

[bib134] Zavarzina DG , Zhilina TN, Kostrikina NA *et al*. *Isachenkonia alkalipeptolytica* gen. nov. sp. nov., a new anaerobic, alkaliphilic proteolytic bacterium capable of reducing Fe(III) and sulfur. Int J Syst Evol Microbiol. 2020;70:4730–8. 32697189 10.1099/ijsem.0.004341

[bib102] Zavarzina DG , Zhilina TN, Kuznetsov BB et al. *Natranaerobaculum magadiense* gen. nov., sp. nov., an anaerobic, alkalithermophilic bacterium from soda lake sediment. Int J Syst Evol Microbiol. 2013;63:4456–61. 23859946 10.1099/ijs.0.054536-0

[bib103] Zeng X , Zhang Z, Li X et al. *Caloranaerobacter ferrireducens* sp. nov., an anaerobic, thermophilic, iron (III)-reducing bacterium isolated from deep-sea hydrothermal sulfide deposits. Int J Syst Evol Microbiol. 2015;65:1714–8. 25736413 10.1099/ijs.0.000165

[bib105] Zhang J , Yang G, Zhou S et al. *Fontibacter ferrireducens* sp. nov., an Fe(III)-reducing bacterium isolated from a microbial fuel cell. Int J Syst Evol Microbiol. 2013;63:925–9. 22659503 10.1099/ijs.0.040998-0

[bib106] Zhilina TN , Zavarzina DG, Detkova EN et al. *Fuchsiella ferrireducens* sp. nov., a novel haloalkaliphilic, lithoautotrophic homoacetogen capable of iron reduction, and emendation of the description of the genus *Fuchsiella*. Int J Syst Evol Microbiol. 2015;65:2432–40. 25908709 10.1099/ijs.0.000278

[bib135] Zhilina TN , Zavarzina DG, Kolganova TV *et al*. *Alkaliphilus peptidofermentans* sp. nov., a new alkaliphilic bacterial soda lake isolate capable of peptide fermentation and Fe(III) reduction. Microbiology. 2009;78:445–54. 19827715

[bib49] Zillig W , Stetter KO, Wunderl S et al. The *Sulfolobus*-“Caldariella” group: Taxonomy on the basis of the structure of DNA-dependent RNA polymerases. Arch Microbiol. 1980;125:259–69.

