# Supplementary material for: Limitations of microbial iron reduction under extreme conditions
Source: FEMS Microbiol Rev. 2022 Jul 16;46(6):fuac033. doi: 10.1093/femsre/fuac033 (PMC9629499; doi:10.1093/femsre/fuac033)
Supplement: fuac033_Supplemental_Files [file fuac033_supplemental_files.zip › Supplementary_Information_130622.docx]

**Supplementary Information**

Iron reduction equations used for thermodynamic calculations

**Acetate (8 electrons transferred in all cases)**

4Fe_2_O_3_ + CH_3_COO^-^ + 15H^+^ ⇌ 8Fe^2+^ + 8H_2_O + 2HCO_3_^-^

8FeO(OH) + CH_3_COO^-^ + 15 H^+^ ⇌ 8Fe^2+^ + 12H_2_O + 2HCO_3_^-^

8Fe(OH)_3_ + CH_3_COO^-^ + 15 H^+^ ⇌ 8Fe^2+^ + + 20H_2_O + 2HCO_3_^-^

8Fe^3+^ + CH_3_COO^-^ + 4H_2_O ⇌ 8Fe^2+^ + 9H^+^ +2HCO_3_^-^

**Hydrogen (2 electrons transferred with hematite and ferrihydrite, 1 with goethite and aqueous Fe(III))**

Fe_2_O_3_ + H_2_ + 4H^+^ ⇌ 2Fe^2+^ + 3H_2_O

FeO(OH) + H_2_ + 2H^+^ ⇌ Fe^2+^ + 2H_2_O

2Fe(OH)_3_ + H_2_ + 4H^+^ ⇌ 2Fe^2+^ + 6H_2_O

2Fe^3+^ + H_2_ ⇌ Fe^2+^ + 2H^+^

Where: Fe_2_O_3_ is haematite; FeOOH is goethite; Fe(OH)_3_ is ferrihydrite; Fe^3+^ is ferric iron ions

Values used in thermodynamic equations

| Species | Formula | Enthalpy  (kJ mol^-1^) | Entropy  (J ⋅ K^-1^) | Gibbs Free Energy (kJ mol^-1^) |
| --- | --- | --- | --- | --- |
| Acetate | CH_3_COO^-^ | -486.8^1^ | 86.6^2^ | -369.41^3^ |
| Hydrogen | H_2_ | 0.0^4^ | 130.6^5^ | 0.00^3^ |
| Ferric Iron | Fe^3+^ | -48.5^5^ | -316.0^5^ | -4.60^5^ |
| Ferrihydrite | Fe(OH)_3_ | -823.0^6^ | 106.7^6^ | -659.4^5^ |
| Goethite | FeO(OH) | -559.3^5^ | 60.5^5^ | -488.60^5^ |
| Haematite | Fe_2_O_3_ | -824.6^5^ | 87.4^5^ | -742.70^5^ |
| Water | H_2_O | -285.8^5^ | 70.0^5^ | -237.18^5^ |
| Bicarbonate ion | HCO_3_^-^ | -692.0^5^ | 91.2^5^ | -586.80^5^ |
| Ferrous Iron | Fe^2+^ | -89.1^5^ | -138.0^5^ | -78.87^5^ |
| Hydrogen ion | H^+^ | 0.0^5^ | 0.0^5^ | 0.00^5^ |

^1^Hammes, G. G. & Hammes-Schiffer, S., 2015. Appendix 4. In: G. G. Hammes & S. Hammes-Schiffer, eds. *Physical Chemistry for the Biological Sciences.* Hoboken, New Jersey: Wiley, pp. 465-466.

^2^Marcus, Y. & Loewenschuss, A., 1984. Chapter 4. Standard entropies of hydration of ion. *Annual Reports Section "C" (Physical Chemistry),* Volume 81, pp. 81-135.

^3^Thauer, R. K., Jungermann, K. & Decker, K., 1977. Energy Conservation in Chemotrophic Anaerobic Bacteria. *Bacteriological Reviews,* 41(1), pp. 100-180.

^4^Dean, J. A., 1979. *Lange's Handbook of Chemistry.* 12th ed. New York, New York: McGraw-Hill.

^5^Stumm, W. & Morgan, J. J., 1996. *Aquatic Chemistry.* 3rd ed. New York, New York: John Wiley & Sons, Inc..

^6^Krauskopf, K. B. & Bird, D. K., 1995. *Introduction to Geochemistry.* 3rd ed. Singapore: Mcgraw-Hill.
